# Genomic differentiation and conservation relevance of isolated marginal *Primula farinosa* populations in Central-Eastern Europe

**DOI:** 10.3389/fpls.2026.1944724

**Published:** 2026-10-09

**Authors:** Nayna Babar, Levente Laczkó, Judit Cservenka, Szilárd Póliska, Nozim Shodiev, András Mészáros, Attila Molnár V., Gábor Sramkó

**Affiliations:** 1 Evolutionary Genomics Research Group, Department of Botany, Institute of Biology and Ecology, University of Debrecen, Debrecen, Hungary; 2 Department of Bioinformatics, One Health Institute, Faculty of Health Sciences, University of Debrecen, Debrecen, Hungary; 3 HUN-REN–UD Conservation Biology Research Group, University of Debrecen, Debrecen, Hungary; 4 Herman Ottó Institute, Budapest, Hungary; 5 Department of Biochemistry and Molecular Biology, Faculty of Medicine, University of Debrecen, Debrecen, Hungary; 6 Balaton-felvidéki National Park Directorate, Csopak, Hungary

**Keywords:** allelic richness, Balaton Region, calcareous fen, cold-adapted species, conservation genomics, phylogeography, RADseq, regional differentiation

## Abstract

**Introduction:**

Quaternary climatic oscillations produced heterogeneous range dynamics in European cold-adapted plants. *Primula farinosa* L. has been discussed in the context of the interglacial expansion model for moisture-demanding cold-adapted species. However, it remains unclear whether isolated low-elevation populations in Central European calcareous fens and wet meadows represent long-term relicts or recently established populations.

**Methods:**

We used RADseq-derived SNPs from four sampling localities in the Balaton Region of Hungary and one low-elevation comparative locality at Lipce in the Julian Alps, Slovenia, to assess population-genomic structure, differentiation, allelic diversity and demographic history. Population structure was analyzed using SNP-based maximum-likelihood tree inference, PCA, model-based ancestry clustering and pairwise F_ST_ estimates. We further quantified rarefied allelic richness and private allelic variation and performed exploratory population-specific historical effective population-size trajectories from folded site-frequency spectra.

**Results:**

Population structure analyses revealed pronounced differentiation between Lipce and the Hungarian samples. Within Hungary, the two neighbouring localities of Lesencetomaj and Tapolca were nearly indistinguishable, whereas Köveskál and Nemeshany populations showed stronger differentiation and reduced allelic diversity. Rarefied allelic richness was highest in Lipce and Lesence and lowest in Nemeshany, with Köveskál only slightly richer. Collectively, the Balaton Region carried a distinct allelic pattern and comparable rarified richness at the broader regional scale. Demographic reconstructions suggested population-specific effective population-size changes over recent to Late-Glacial timescales, although older intervals were weakly resolved and exact timing was uncertain.

**Discussion:**

Low-elevation *Primula farinosa* populations in Hungary and Slovenia are strongly differentiated, and this isolation is unlikely to be explained solely by recent anthropogenic wetland fragmentation. The higher rarefied allelic richness of Lipce and Lesence suggests that these populations retain less-eroded genetic variation, whereas the depauperate allelic profiles of Köveskál and especially Nemeshany are consistent with recent founder effects, bottlenecks, strong drift or long-term small effective population size. These findings indicate that the Balaton Region populations represent a conservation-relevant and regionally differentiated component of *P. farinosa* genomic diversity. Although the exact timing and historical origin of their isolation remain unresolved, the observed genomic pattern is compatible with, but does not demonstrate, long-term regional persistence, including a possible Late Glacial refugial scenario.

## 1 Introduction

The effects of Quaternary glacial-interglacial cycles on European plant distributions have often been interpreted through the classical temperate-refugia paradigm (Hewitt, 1999). Under this model, many temperate taxa persisted during glacial maxima in southern refugia, especially in the Iberian, Italian and Balkan peninsulas, and subsequently expanded northwards after climatic amelioration (Hewitt, 2004; Tzedakis et al., 2013). This paradigm has been highly influential, but it does not describe the histories of cold-adapted taxa. Species associated with arctic, boreal, montane or cold wetland habitats, often termed as ‘cold-adapted’, may respond idiosyncratically to climatic oscillations because their distributions are constrained not only by temperature, but also by growing-season length, moisture, soil chemistry, competition and the availability of suitable habitats (Birks, 2008; Crawford, 2008; Stewart et al., 2010).

Two main hypotheses have been proposed for cold-adapted species during Late Quaternary climatic cycles. The better-established interglacial contraction hypothesis assumes that cold-adapted taxa expanded during glacial periods into extensive ice-free peripheral, low-elevation or lowland tundra-steppe habitats, and subsequently contracted during interglacials into areas where suitable cool or moist conditions persisted, including northern regions, mountain systems and locally favourable refugial habitats (Hewitt, 2004; Stewart et al., 2010). This scenario is supported by several studies of alpine and arctic organisms, including European high-mountain plants, arctic-alpine invertebrates and cold-adapted vertebrates, which show patterns consistent with larger glacial distributions followed by postglacial or interglacial retreat and fragmentation (Kropf et al., 2003; Muster and Berendonk, 2006; Dalén et al., 2007; Westergaard et al., 2011; Espíndola et al., 2012). Under this model, present-day disjunctions are interpreted mainly as remnants of formerly broader cold-stage ranges.

The alternative interglacial expansion hypothesis proposes a different demographic mechanism. Some cold-adapted species may not have expanded widely during full glacial conditions, despite the colder climate, because suitable habitats were not determined by temperature alone. For moisture-demanding, weakly competitive plants, the availability of persistently wet, open and calcareous habitats may have been more limiting than regional temperature, especially where full-glacial landscapes were dominated by open, continental and often dry vegetation mosaics (Schmitt, 2007; Harrison and Prentice, 2003; Fletcher et al., 2010; Magyari et al., 2010). According to this hypothesis, such species persisted during glacial maxima in relatively moist low-elevation, peripheral or near-glacial refugia, and expanded during warmer interglacials when retreating glaciers exposed new open habitats and when suitable wet habitats became available at higher altitudes and latitudes (Birks, 2008; Stewart et al., 2010). This mechanism is expected particularly for cold-adapted species with broad altitudinal or ecological amplitude, rather than strict high-alpine specialists, and for taxa whose present ranges combine Central European mountain occurrences with southern-boreal or lowland northern occurrences (Ehrich et al., 2008; Lienert et al., 2002; Oliver et al., 2006; Ronikier et al., 2008; Šmídová et al., 2011; Urbaniak and Kwiatkowski, 2023).

The biological logic of the interglacial expansion model is especially relevant for wetland and fen specialists. During the Holocene, warming and the spread of temperate forests and tall-herb vegetation likely increased competition in many low-elevation landscapes. Cold-adapted plants with low competitive ability could therefore persist outside mountains only where edaphic or hydrological conditions maintained open vegetation, for example in calcareous fens, river gravels, cliffs or other treeless refugial habitats (Birks, 2008; Hájek et al., 2006, Hájek et al., 2011). The key point is that low-elevation populations of cold-adapted plants are not automatically ecological anomalies or recent sink populations. In some cases, they may represent remnants of formerly broader distributions, preserved by local habitat conditions while surrounding landscape became climatically or competitively unsuitable.

*Primula farinosa* L. is key empirical system in which the interglacial expansion hypothesis has been proposed to explain present-day distribution and genomic diversity (Theodoridis et al., 2017). It is a cold-adapted, insect-pollinated, heterostylous and mostly outcrossing perennial associated with moist calcareous habitats, including calcareous fens, wet meadows, lake and stream margins and, at higher elevations, calcareous grasslands (Hambler and Dixon, 2003; Richards, 2003). In southern Europe, including the Alps, Carpathians and Pyrenees, it is primarily a montane to alpine species, whereas in northern Europe it also occurs in low-elevation flatlands (Hambler and Dixon, 2003; Richards, 2003; Theodoridis et al., 2013). Its broad, fragmented European distribution and its inferred Late Quaternary evolutionary history make it a particularly informative system for testing how cold-adapted plants responded to climatic cycles (de Vos et al., 2014; Stewart et al., 2010).

Theodoridis et al. (2017) explicitly tested the interglacial expansion hypothesis in *P. farinosa* by integrating species distribution models projected across the last 72 thousand years (ky) with genome-wide reduced-representation sequence data and approximate Bayesian computation. Their analyses supported a distribution-wide interglacial expansion rather than the classical expectation of broader glacial distributions followed by interglacial contraction in cold-adapted taxa. They also argued that this result is biologically plausible because *P. farinosa* has broader climatic preferences than several closely related alpine/arctic primroses, prefers wet open microhabitats, and is a weak competitor; consequently, it may have been constrained by dry glacial steppes but favoured by the availability of open, moist habitats after glacial retreat (Lindborg and Ehrlén, 2002; Theodoridis et al., 2013, Theodoridis et al., 2017).

The Hungarian low-elevation occurrences of *P. farinosa* are especially relevant in this framework. In the Carpathian Basin, including Hungary, vegetation reconstructions indicate dry, *Artemisia*-rich steppes at low altitudes during the Last Glacial Maximum (LGM) (Magyari et al., 2010; Feurdean et al., 2014; Magyari et al., 2014). Yet, the species has long-known natural occurrences (Borbás, 1900) from fen meadows in the Balaton Region (incl. the Balaton Uplands), where persistence depends on very specific wet, open, calcareous microhabitats (Salamon-Albert and Morschhauser, 2003). These occurrences are commonly reported as ‘glacial relicts’ in the local literature (Borbás, 1900; Borhidi et al., 2012). However, such terminology requires careful evaluation, because present-day occurrence in isolated fen habitats does not by itself establish uninterrupted glacial persistence. Central European calcareous fens are groundwater-fed, nutrient-limited habitats with distinctive plant and bryophyte assemblages, and several fen specialists show distributions consistent with long-term regional persistence or Holocene-scale relict dynamics (Hájek et al., 2006, Hájek et al., 2011).

This relict interpretation raises a testable population-genomic expectation and defines the contribution of the present study. If Balaton Region populations represent long-term relicts (i.e., preglacial or glacial) or remnants of older regional persistence, they may be expected to retain genomic signatures often associated with ‘rear edge’ populations (Hampe and Petit, 2005), such as strong differentiation, reduced contemporary connectivity, and population-specific components of allelic diversity. Conversely, if they are recent Holocene colonists or introductions, they may show patterns more consistent with recent founder events, reduced allelic richness and limited private variation. Because our sampling includes only one comparative low-elevation Alpine-region population, Lipce is treated as a regional comparative reference for evaluating the distinctiveness of the Balaton Region populations.

By examining these alternatives with RADseq-derived SNPs from five Central European populations, the study extends the species-wide interglacial-expansion framework for *Primula farinosa* to a finer regional scale and evaluates whether isolated low-elevation fen populations preserve distinct components of genomic diversity relevant for conservation. Specifically, we asked: (i) how genomic variation is structured among the Balaton Region populations and the comparative Lipce population; (ii) whether allelic variability and genomic differentiation are compatible with long-term regional persistence, recent colonization or strong drift in isolated populations; and (iii) whether site-frequency-spectrum-based demographic reconstruction reveals population-specific changes in effective population size, while acknowledging the limited ability of these data to resolve the exact timing of population origin or isolation.

## 2 Materials and methods

### 2.1 Sampling and study populations

RADseq data were generated for 47 individuals assigned to five sampling localities: Lipce, Köveskál, Nemeshany, Tapolca: Körtvélyes and Lesencetomaj: Sövényháza (**Figure 1**; **Table 1**). These localities were initially treated as separate population units in the bioinformatic analyses, with the labels Lip, Kov, Nem, Kor and Sor, respectively. Lipce was included as a low-elevation comparative locality from the Alpine region, occurring in marshland habitat in the Julian Alps, Slovenia. All other localities represent isolated low-elevation occurrences of *P. farinosa* in the Balaton Region, Hungary, traditionally interpreted as glacial relicts in the local floristic literature. Tapolca: Körtvélyes and Lesencetomaj: Sövényháza are located in the same headwater basin of the Lesence Stream in close geographic proximity, and they are separated by an approximately 0.5 km of unsuitable habitat, including a paved road. They were therefore initially analysed as separate sampling localities to test whether genetic differentiation was detectable at this fine spatial scale. Because population genomic analyses showed little or no differentiation between them, they were subsequently combined and treated as a single Lesence biological population in downstream analyses. Analyses aimed at describing fine-scale structure retained the original Körtvélyes and Sövényháza labels.

**Figure 1:**
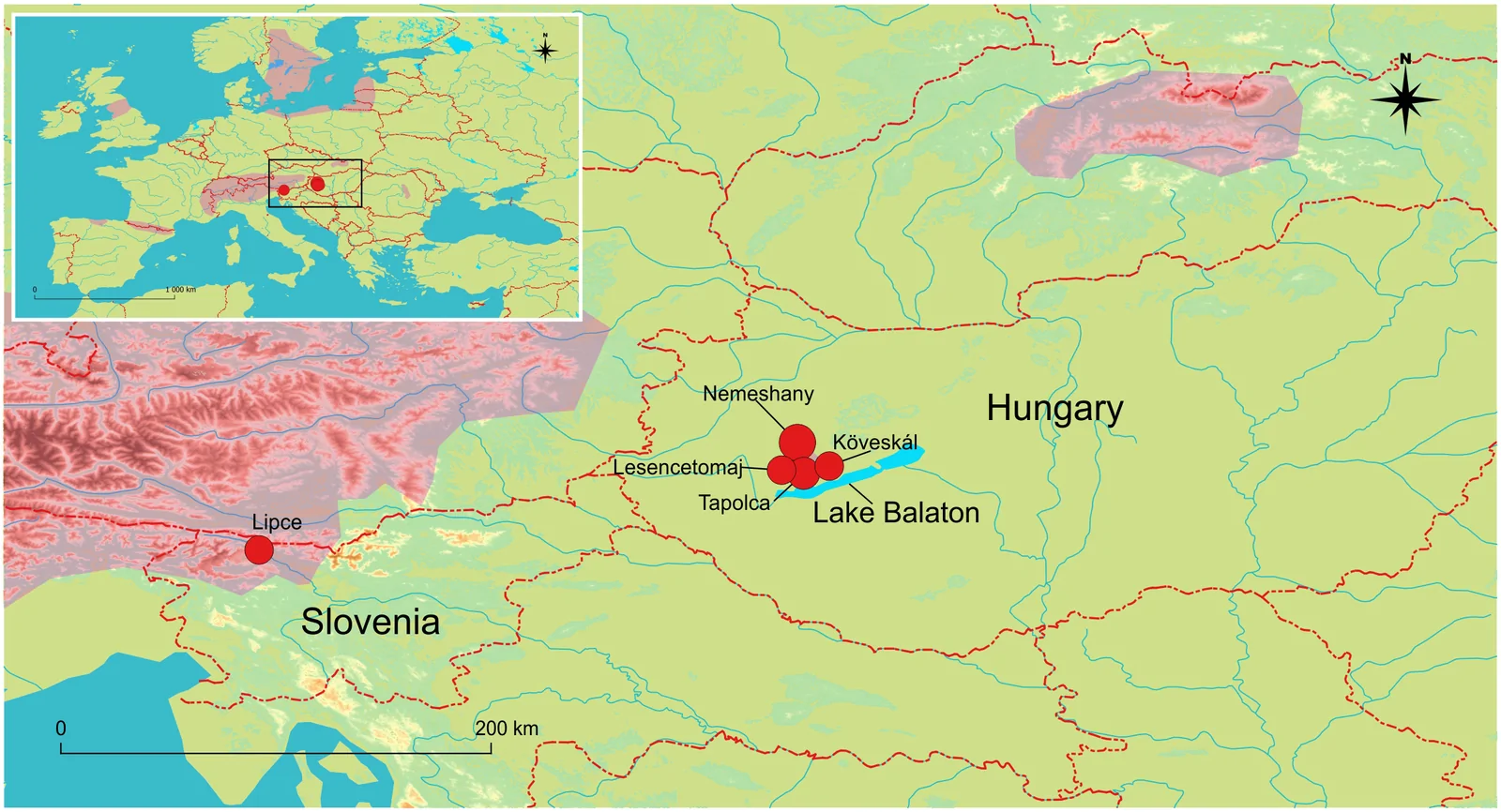
Geographic position of the sampled *Primula farinosa* populations. The inset shows the approximate current European distribution of *P. farinosa* and the location of the study region; the main map shows the Slovenian comparative population at Lipce and the Balaton Region populations in Hungary. Dot size corresponds to sample size. The shaded area indicates the approximate current distribution of *P. farinosa* based on Theodoridis et al. (2017).

**Table 1:** Geographic origin and sample sizes (N) of *Primula farinosa* populations included in the study.

Population	N	Locality	Coordinates (N, E)	Altitude
Lipce	8	SI: Julian Alps: Lipce	46.4, 14.07	607 m
Köveskál	8	HU: Balaton Region: Köveskál	46.88, 17.6	174 m
Nemeshany	13	HU: Balaton Region: Nemeshany	47.06, 17.38	152 m
Körtvélyes	10	HU: Balaton Region: Tapolca	46.87, 17.4	143 m
Sövényháza	8	HU: Balaton Region: Lesencetomaj	46.87, 17.38	144 m

Country name abbreviation follows ISO-2 standards. Decimal coordinates are rounded for conservation reasons.

*Primula farinosa* is a strictly protected species in Hungary, and the field sampling, which involved the removal of two leaves from each sampled individual, was conducted in the presence of the local nature conservation authority (Balaton-felvidéki National Park Directorate) under the permit issued by the Pest County Government Office (‘Pest Megyei Kormányhivatal’; permit no. PE-KTFO/1568-18/2022). The leaf tissue samples were immediately placed into a resealable sachet filled with approximately 10 g silica gel, and individual coordinates (latitude, longitude) were collected. We aimed to keep at least 5 metres between the sampled individuals, and we also endeavoured to spatially cover the full extent of the population’s area to represent the variability of the populations in the analyses.

### 2.2 DNA extraction and RADseq library preparation

Total genomic DNA was extracted from silica-dried leaf material using a modified CTAB protocol based on Sramkó et al. (2014). Leaf fragments were homogenized with sterile sand and polyvinylpolypyrrolidone (PVPP), and the powder was incubated in 2% CTAB extraction buffer containing β-mercaptoethanol and polyvinylpyrrolidone (PVP). Proteins and other contaminants were removed by ammonium acetate treatment, followed by chloroform:isoamyl alcohol purification. DNA was then recovered by precipitation with 96% ethanol, the pellets were rinsed twice in 70% ethanol, and the final DNA was dissolved in 10 mM Tris-HCl (pH 8.0). Isolate quality was first checked on 1% agarose gels using standard electrophoresis. DNA concentration and purity were then estimated by Qubit v3 fluorometry (Thermo Fisher Scientific Ltd., Waltham, MA, USA) and NanoDrop Lite v3 spectrophotometry. Only high-molecular-weight, visually intact, DNA extracts with a fluorometric/spectrophotometric concentration ratio between 0.9 and 1.4 were retained for library construction.

RADseq libraries were prepared according to a classical single-digest RAD protocol (Baird et al., 2008) using PstI (CTGCA/G) (New England Biolabs, Ipswich, MA, USA) as the restriction enzyme. Custom-modified Illumina TruSeq P1 adapters were ligated to the restriction overhangs, after which the DNA was fragmented using a Bioruptor Pico instrument (Diagenode SA, Seraing, Belgium) with five cycles of 30 s sonication at high power followed by 30 s pauses. Fragment pools were subjected to double-sided size selection with the SPRI Select Beads Kit (Beckman Coulter Inc., Brea, CA, USA) using SPRI beads in ratios of 0.65× and 0.7×. Custom-modified P2 adapters were subsequently ligated with T4 DNA ligase (New England Biolabs). Library preparation was carried out in sub-libraries of 12 samples, which were then pooled in equimolar proportions. Adapter-ligated fragments were amplified with Phusion High-Fidelity PCR Master Mix with HF Buffer (New England Biolabs); PCR artifacts were reduced by limiting amplification to 14 cycles and by carrying out eight replicate reactions that were pooled afterwards. The pooled library was size-selected again with SPRI beads using a bead ratio of 0.7×, targeting an insert distribution around 400 bp. Final library size profile and concentration were assessed on a Bioanalyzer system (Agilent Technologies Inc., Santa Clara, CA, USA). Sequencing was performed at the Genomic Medicine and Bioinformatics Core Facility of the University of Debrecen, Hungary, on an Illumina NextSeq 500 platform, generating 150-bp single-end reads.

### 2.3 RADseq processing, assembly optimization and SNP discovery

Raw RADseq reads were demultiplexed and quality filtered with process_radtags in Stacks v2.2 (Catchen et al., 2013). Reads were assigned to samples using inline barcodes and PstI as the restriction enzyme. Data were processed as Phred+33 FASTQ files with barcode rescue, standard cleaning and quality filtering enabled. Reads were truncated to 140 bp, and reads exceeding the length limit were discarded. Initial read quality was assessed with the FastQC software tool, followed by adapter removal and base-level quality filtering (Q ≤ 5) using fastp v1.0.1 (Chen et al., 2018).

In the lack of a reference genome for *Primula farinosa*, we applied a *de novo* RAD locus assembly and SNP calling approach using the denovo_map.pl module of Stacks. Before final SNP discovery, assembly parameters were optimized following the parameter-space exploration strategy proposed for RADseq datasets (Paris et al., 2017). Optimization was performed on ten representative samples by varying the minimum depth required to create a stack within individuals (m = 3–6) and the maximum number of mismatches allowed within individuals and during catalogue construction (M = n = 2–6). For each parameter combination, denovo_map.pl was run and the populations module was used to export loci present in at least 80% of individuals. Parameter combinations were compared using the number of retained loci and SNPs, sample coverage, total retained loci and individual missingness. This screening identified m = 3, M = 5 and n = 5 as the most appropriate setting for the final *de novo* assembly.

The final *de novo* assembly was therefore performed in Stacks using denovo_map.pl with m = 3, M = 5 and n = 5. SNPs were exported with the populations module, requiring loci to be present in at least four populations (-p 4). The raw VCF file generated by Stacks was filtered using vcftools v0.1.16 (Danecek et al., 2011). Sites with more than 20% missing data were removed, and the dataset was restricted to biallelic SNPs. To reduce the effect of physical linkage, one SNP was randomly selected from each retained RAD locus after filtering using a custom bash script. In the final filtering step, SNPs were retained only if mean read depth across individuals was between 5 and 60. The resulting one-SNP-per-RAD-locus dataset contained biallelic SNPs present in at least four populations, with no more than 20% missing data, mean depth between 5 and 60, and at most one randomly selected SNP per RAD locus. This dataset was used for population structure, pairwise F_ST_, allelic-richness and private-allele analyses.

### 2.4 Maximum-likelihood SNP-based tree inference

Maximum-likelihood phylogenetic tree reconstruction was performed in IQ-TREE v2.4.0 using the final filtered one-SNP-per-RAD-locus dataset (Minh et al., 2020). Because the alignment contained only variable positions, ascertainment-bias correction was applied during model selection and tree inference following the ASC approach for variable-only data (Lewis, 2001). The best-fitting model was selected using ModelFinder under MFP+ASC according to the Bayesian information criterion (Kalyaanamoorthy et al., 2017) as implemented in IQ-TREE. Nodal support was evaluated with 1,000 ultrafast bootstrap replicates and 1,000 SH-like approximate likelihood-ratio test replicates (Anisimova and Gascuel, 2006; Hoang et al., 2018).

### 2.5 Population structure analyses

Principal component analysis was performed in PLINK v1.9.0-b.8 (Chang et al., 2015) using the final filtered one-SNP-per-RAD-locus dataset (Purcell et al., 2007). To ensure compatibility with PLINK, all SNPs were assigned to a single artificial chromosome and given unique identifiers based on their original scaffold and position. The VCF was converted to binary PLINK format using --make-bed and --double-id, after which the first 20 principal components were calculated. Eigenvectors and eigenvalues were imported into R, where the percentage of variance explained by each principal component was calculated. The first two principal components were visualized in R using ggplot2 and the ggrepel package for non-overlapping text labels, with samples colored by population and labeled by individual number (Wickham, 2016).

Ancestry clustering was performed with ADMIXTURE v1.3.0 (Alexander et al., 2009) using the final filtered one-SNP-per-RAD-locus dataset. Analyses were run for K values from 1 to 5, corresponding to the range from a single inferred ancestry component to the number of sampled populations. The preferred K was identified using cross-validation error, while higher K values were inspected as exploratory summaries of hierarchical structure rather than as independently confirmed demographic units.

A NeighborNet network was generated in SplitsTree v6.5.1 (Huson and Bryant, 2024) to provide a supplementary visualization of genetic relationships and potential conflicting signal among individuals. Pairwise genetic distances were calculated in SplitsTree using uncorrected *p*-distances, and the NeighborNet algorithm (Bryant and Moulton, 2004) was used to construct the network. The resulting network was interpreted as a distance-based visual summary of population structure.

### 2.6 Pairwise F_ST_ and clustering based on genetic differentiation

Pairwise genetic differentiation among populations was quantified using Weir and Cockerham’s estimator of F_ST_ (Weir and Cockerham, 1984) as implemented in vcftools. Calculations were based on the final filtered one-SNP-per-RAD-locus dataset. Population-specific sample lists were supplied to vcftools using the --weir-fst-pop option, and all pairwise comparisons were performed among the five analyzed populations. For each comparison, the weighted Weir and Cockerham F_ST_ estimate was extracted from the output and arranged into a symmetric pairwise F_ST_ matrix. To assess uncertainty around pairwise differentiation estimates, we additionally used the site-wise Weir and Cockerham F_ST_ values reported by VCFtools and performed non-parametric bootstrap resampling across SNPs/loci. Because the final dataset contained one randomly selected SNP per RAD locus, SNP-based resampling approximated locus-level resampling. For each population pair, 10,000 bootstrap replicates were generated by sampling SNPs with replacement, and 95% confidence intervals were calculated from the 2.5% and 97.5% percentiles of the bootstrap distribution. To visualize relationships among populations based on genetic differentiation, the resulting pairwise F_ST_ matrix was imported into PAST v4.17 and used as a user-supplied distance matrix for UPGMA clustering (Hammer et al., 2001). The resulting dendrogram was interpreted as a phenetic summary of relative genetic differentiation among populations, rather than as an explicit demographic or phylogenetic model.

### 2.7 Allelic richness and private alleles

To complement the population-structure and demographic analyses, we quantified rarefied allelic richness and private allelic variation from the filtered SNP dataset. The VCF file was imported into R v4.5.1 using the vcfR v1.13.0 package (Knaus and Grünwald, 2017) and converted to a genind object with adegenet v2.1.10 (Jombart, 2008). Rarefied allelic richness was estimated using the allelic.richness function of hierfstat v. 0.5-11 (Goudet, 2005) after conversion of the genind object to hierfstat format. Allelic richness was calculated for each locus and population, and mean allelic richness per population was obtained by averaging locus-specific estimates across loci while excluding missing values. This rarefaction-based approach allowed comparison of allelic richness among populations while accounting for differences in sample size (El Mousadik and Petit, 1996; Kalinowski, 2004). Uncertainty around mean rarefied allelic richness was assessed by non-parametric bootstrap resampling across loci. For each population and regional group, loci were sampled with replacement 10,000 times, the mean rarefied allelic richness was recalculated for each replicate, and 95% confidence intervals were calculated as the 2.5% and 97.5% percentiles of the bootstrap distribution.

Private alleles were quantified using the private_alleles function of poppr v. 2.9.6 (Kamvar et al., 2014). For each population, we calculated the total number of private alleles, the number of loci carrying at least one private allele and the mean number of private alleles per locus. Because raw private-allele counts are sensitive to sample size and missing data, interpretation emphasized rarefied allelic richness and mean private alleles per locus rather than total private-allele counts alone. Mean private alleles per locus were bootstrapped by resampling loci with replacement 10,000 times, and 95% confidence intervals were calculated from the 2.5% and 97.5% percentiles of the bootstrap distribution.

In addition to population-level analyses, we repeated the same rarefied allelic-richness and private-allele calculations at a broader, regional level. For this analysis, individuals were reassigned according to a regional population map, allowing comparison between the Balaton region and Lipce. The same workflow was then applied.

### 2.8 Historical effective population size reconstruction

Historical changes in effective population size were inferred using Stairway Plot v2.2 (Liu and Fu, 2015, Liu and Fu, 2020) from population-specific folded site-frequency spectra (SFS). Analyses were based on independently generated population-specific SFS datasets. Demographic inference was treated as exploratory because the SFSs were derived from reduced-representation RADseq data, were folded due to the absence of reliable ancestral-state information, and were based on SNPs retained after population-specific locus callability and SNP filtering. Specifically, we used the Stacks populations module v2.2 to generate population-specific VCF files from the final *de novo* assembly (denovo_map.pl run with m = 3, M = 5 and n = 5). For each demographic unit, the populations module was run to restrict the analysis only to non-missing variants (flags: -p 1 and -r 1.0) in all successfully genotyped individuals, using only individuals assigned to the focal population, thereby avoiding the requirement that loci be shared across multiple populations. Folded spectra were generated separately for four populations: Köveskál, Lesence, Nemeshany and Lipce. For each population, the number of chromosomes was set according to the number of successfully genotyped diploid individuals included in the corresponding SFS: 16 chromosomes for Köveskál, 36 for Lesence, 26 for Nemeshany and 16 for Lipce. Population-specific callable sequence lengths from the corresponding population-specific Stacks populations output were used as the L parameter in the Stairway Plot blueprint files: 3,692,520 bp for Köveskál, 2,367,401 bp for Lesence, 3,502,068 bp for Nemeshany and 3,686,762 bp for Lipce. Folded SFSs were supplied as integer counts of segregating sites, and whether_folded was set to true.

Folded site-frequency spectra were generated from these population-specific VCF files produced separately for each demographic unit. At each biallelic SNP, REF and ALT alleles were counted across diploid individuals. Sites with missing genotypes within the focal population were excluded so that each retained SNP contributed the full population-specific number of chromosomes to the SFS. Because ancestral states were unavailable, spectra were folded by assigning each SNP to the minor-allele-count class. Monomorphic sites within a population were not included in the polymorphic SFS line, whereas the total number of callable sites was supplied separately as the L parameter.

Stairway Plot analyses were run independently for each population. The proportion of sites used for training was set to 0.67, and 200 bootstrap input files were generated for each population. The number of random breakpoints was set to 4, 7, 11 and 14, following recommendations for reduced-representation-scale datasets (Liu and Fu, 2020). Demographic histories were scaled using a mutation rate of 5.28 × 10^–8^ mutations per site per generation assessed for our focal species (Theodoridis et al., 2017) and a generation time of 2.5 years. This latter parameter was given as five years for *P. farinosa* by Theodoridis et al. (2017), but our extensive observations of the Hungarian populations suggest a shorter generation time: the first flowering usually takes place in the second, sometimes in the third year. Therefore, we set a generation time of 2.5 years for the analysis. Nevertheless, to evaluate the effect of generation-time uncertainty on the temporal scale of the inferred trajectories, Stairway Plot was also run with a generation time of 5 years as a sensitivity analysis. This analysis was used to assess whether the main qualitative population-specific trajectories were robust to generation-time assumptions, while recognizing that both calendar timing and absolute Ne estimates may vary with scaling parameters. The time axis was plotted in thousands of years before present. Because demographic inference was based on RADseq data and folded spectra, the resulting trajectories were interpreted as broad historical trends in effective population size rather than high-resolution reconstructions of demographic change.

## 3 Results

### 3.1 RADseq assembly and filtered SNP dataset

Demultiplexing yielded 63.3 million reads across 48 samples, with a mean of 1.32 million reads per sample. Sample-level read numbers ranged from 206,467 to 2,150,312 reads, indicating generally high sequencing representation across individuals, although one sample (Kov07) had markedly lower read recovery (16% of the mean read count) and was removed from the dataset. The optimized Stacks assembly retained a large set of RAD loci suitable for population-genomic analysis. The final m3M5n5 assembly retained 66,614 loci from 121,578 assembled loci after applying the requirement that loci be present in at least four populations. Across these loci, 9,741,500 sites were represented, including 194,907 variable sites before downstream filtering. Population-level callable sequence lengths were broadly comparable among populations, ranging from 8,778,221 sites in Lipce to 9,612,655 sites in Nemeshany. After removing sites with more than 20% missing data, restricting the dataset to biallelic SNPs, filtering by mean read depth and retaining a single SNP per RAD locus, the final filtered one-SNP-per-RAD-locus dataset contained 32,628 high-quality variants that were used for all exploratory analyses of population structure, allelic diversity, but not for demographic history. For historical effective population size reconstruction, the starting final m3M5n5 dataset was filtered for 24,673 (Köveskál), 15,012 (Lesence), 20,228 (Lipce), and 14,249 (Nemeshany) biallelic SNPs that were present in all individuals within the population from 3,692,520 bp (Köveskál), 3,686,762 bp (Lipce), 2,367,401 bp (Lesence), and 3,502,068 bp (Nemeshany) callable sites.

### 3.2 Maximum-likelihood tree reconstruction

The maximum-likelihood tree revealed strong population-level structuring (**Figure 2**). When rooted with Lipce as the external comparative population, it was separated from all Hungarian populations by a long internal branch with maximal nodal support. Within the Hungarian samples, Nemeshany and Köveskál formed well-supported, coherent clusters, with only one Köveskál individual (#06) clustering with the Tapolca: Körtvélyes and Lesencetomaj: Sövényháza samples. These latter two populations showed close affinity and extensive intermixing, consistent with their geographic proximity in the Lesence Basin. The Lesence samples were sister to Nemeshany and Köveskál within the Hungarian populations.

**Figure 2:**
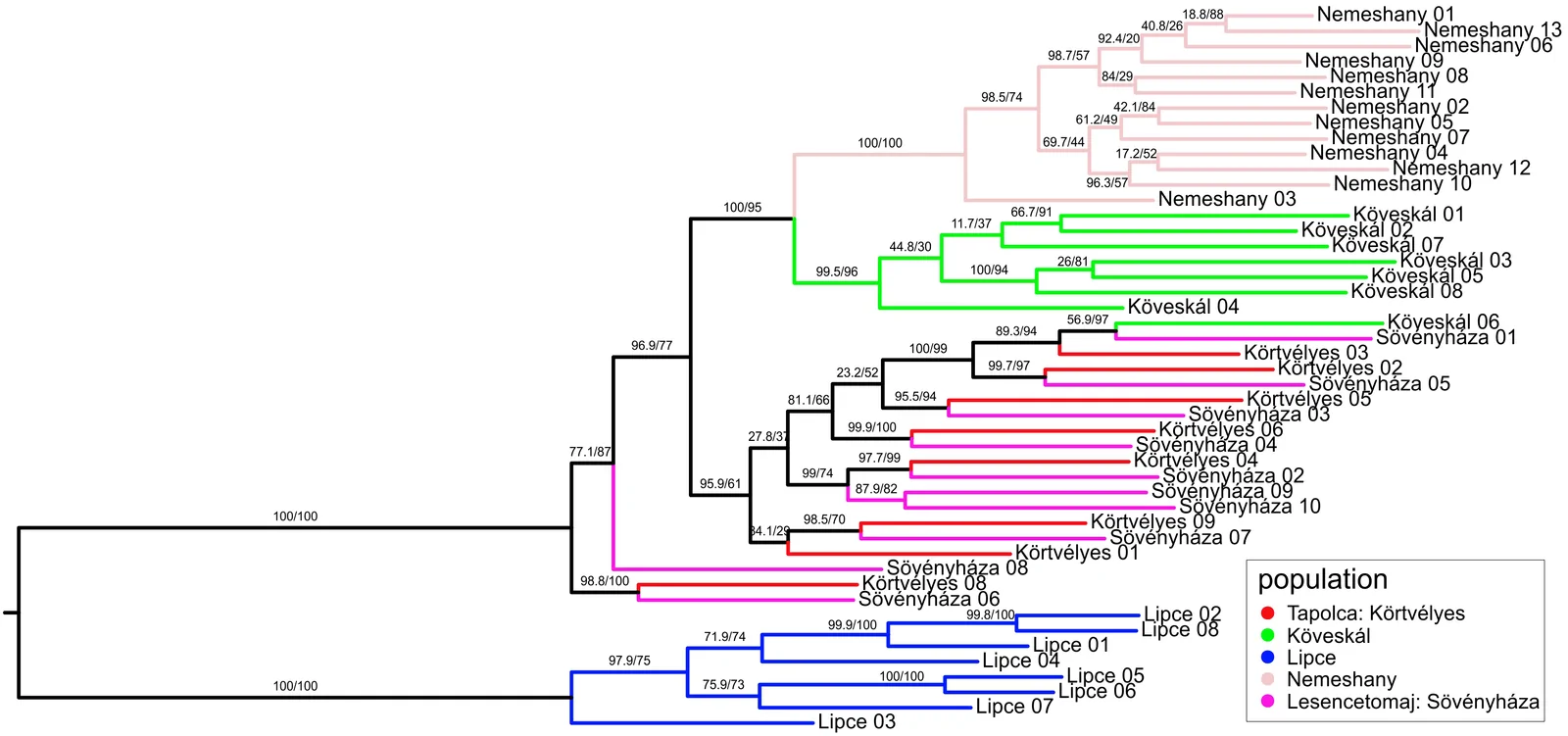
Maximum-likelihood phylogenetic tree reconstruction. IQ-TREE analysis based on 10,096 variable SNP sites. Numbers at nodes indicate SH-like approximate likelihood-ratio test support and ultrafast bootstrap support. Colors denote sampled populations. The tree was rooted on the samples from the comparative outgroup population, Lipce.

### 3.3 Population structure

PCA ordination of allelic variation (**Figure 3**) supported the main relationships inferred from the phylogenetic tree. PC1 explained 19.3% of total variation and clearly separated Lipce from all other populations. PC2 explained 6.3% of total variation and differentiated Nemeshany from the remaining Hungarian populations. Subsequent axes each explained substantially less variation: PC3 explained 5.4%, PC4 5.1% and PC5 4.8%, and the remaining PCs each explained less than 5% of the variation. Köveskál occupied an intermediate but coherent position. In contrast, Tapolca and Lesencetomaj clustered closely together and showed limited intermixing. Again, individual #06 from Köveskál showed affinity to the Lesence group (i.e. Tapolca: Körtvélyes and Lesencetomaj: Sövényháza).

**Figure 3:**
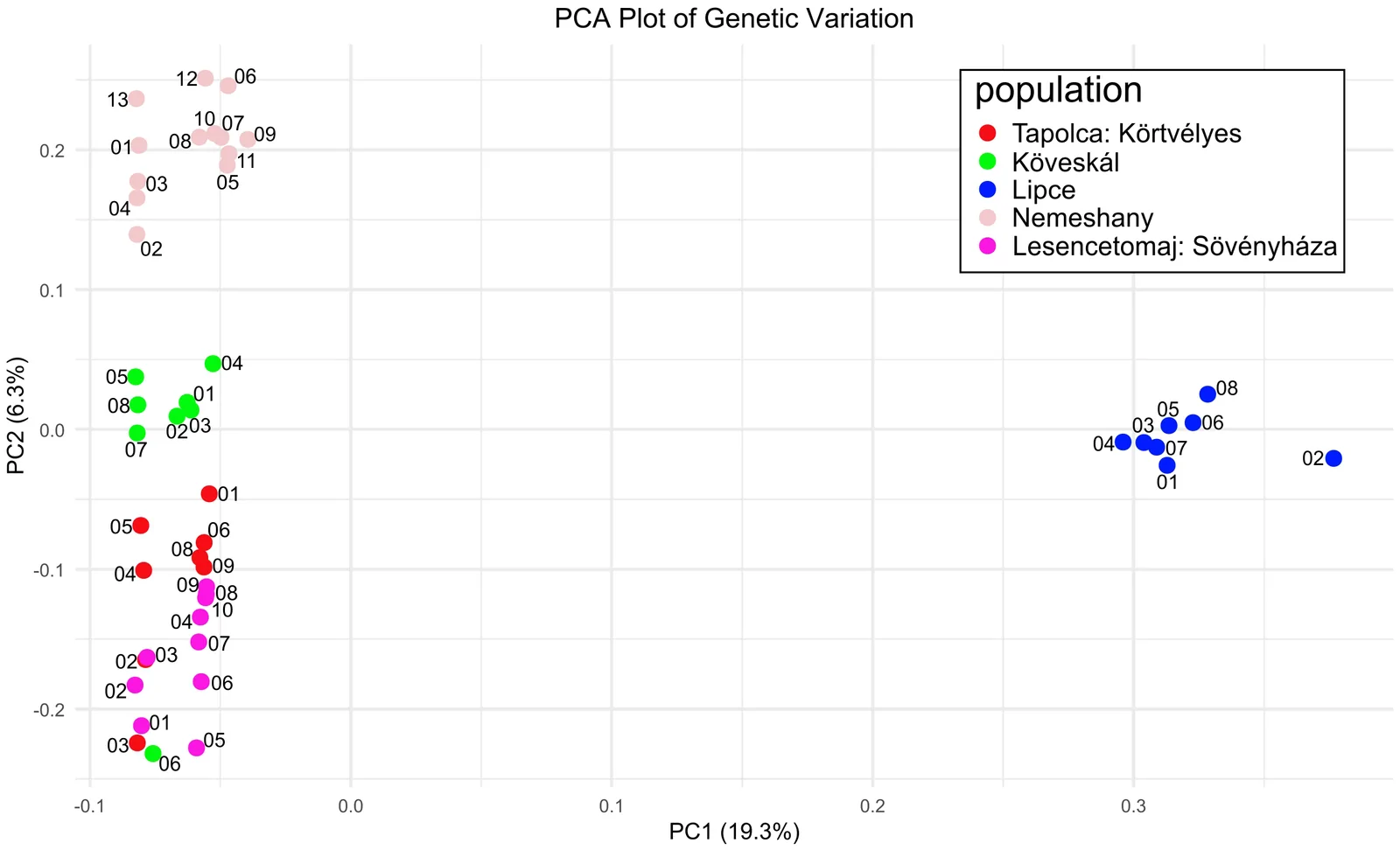
Principal component analysis of RADseq SNP variation. PCA was calculated in PLINK using the filtered one-SNP-per-RAD-locus dataset. PC1 explains 19.3% of variation and PC2 explains 6.3% of variation. Points are colored by population and labeled by individual number.

ADMIXTURE cross-validation identified K = 2 as the best-supported model, with the lowest cross-validation error (CV = 0.38879; **Figure 4A**). Under K = 2, individuals from Lipce were assigned almost entirely to one ancestry component, whereas the Hungarian populations were dominated by the alternative component (**Figure 4B**). Higher values of K revealed additional structure among the Hungarian populations, particularly the separation of Nemeshany and Köveskál, while Körtvélyes and Sövényháza remained closely related. However, these values were inspected as exploratory summaries of hierarchical population structure, but were not interpreted as independently confirmed historical ancestral populations or discrete demographic units.

**Figure 4:**
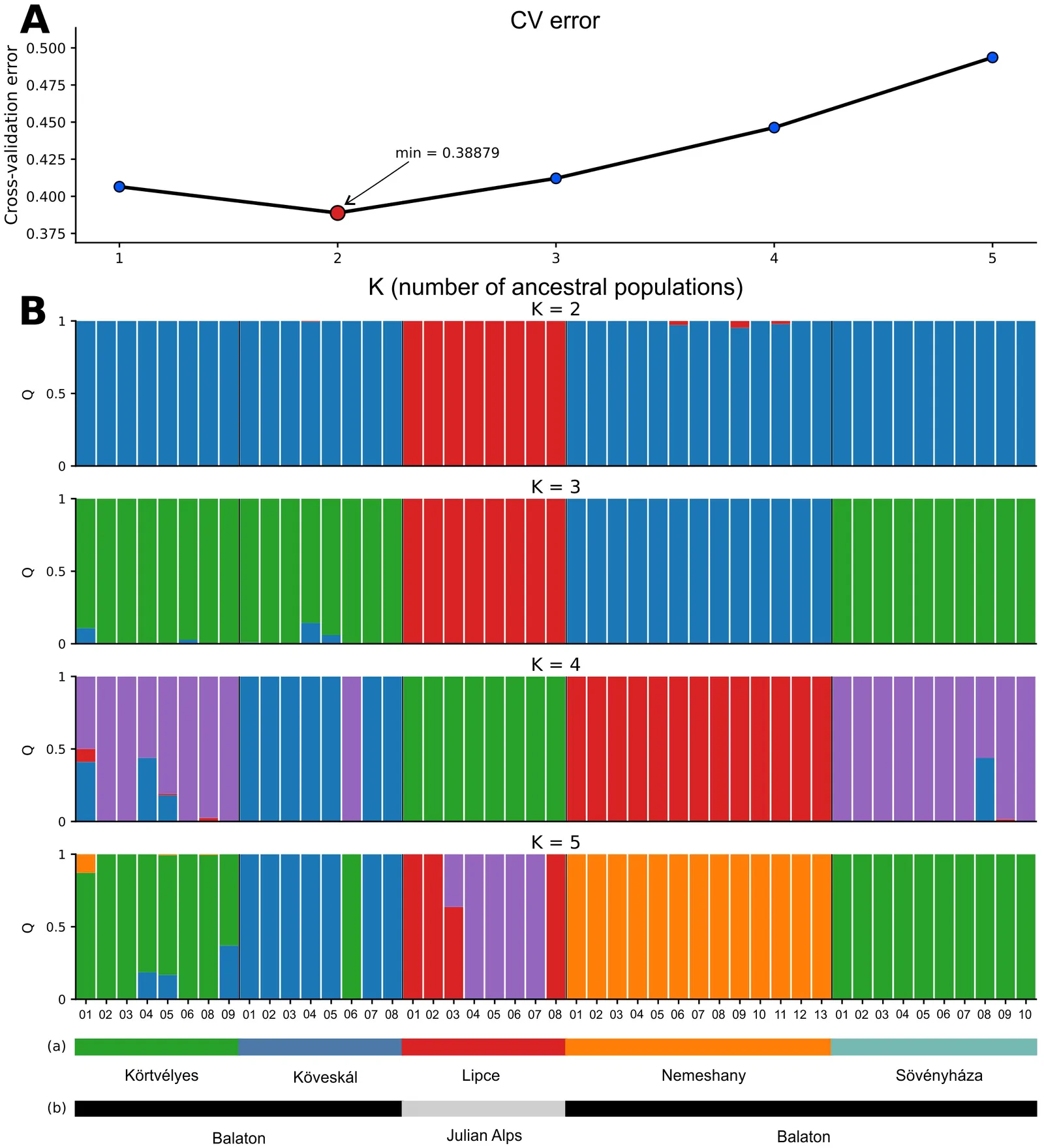
ADMIXTURE analysis. **(A)** Cross-validation error for K = 1–5, with the lowest value at K = 2. **(B)** Individual ancestry coefficients for K = 2–5. Samples are ordered by population and geographic grouping.

A NeighborNet analysis provided a concordant visual summary of genetic relationships with a high fit value (99.6). This high fit indicates that the displayed split network captured nearly all variation in the pairwise distances among individuals. Lipnice individuals are clearly separated from all Hungarian samples by a long split, while the Hungarian samples formed a more compact, reticulate cluster, with Köveskál and Nemeshany showing differentiation, while the Lesence samples — Körtvélyes and Sövényháza — are more intermingled around the central part of the network (**Figure 5**).

**Figure 5:**
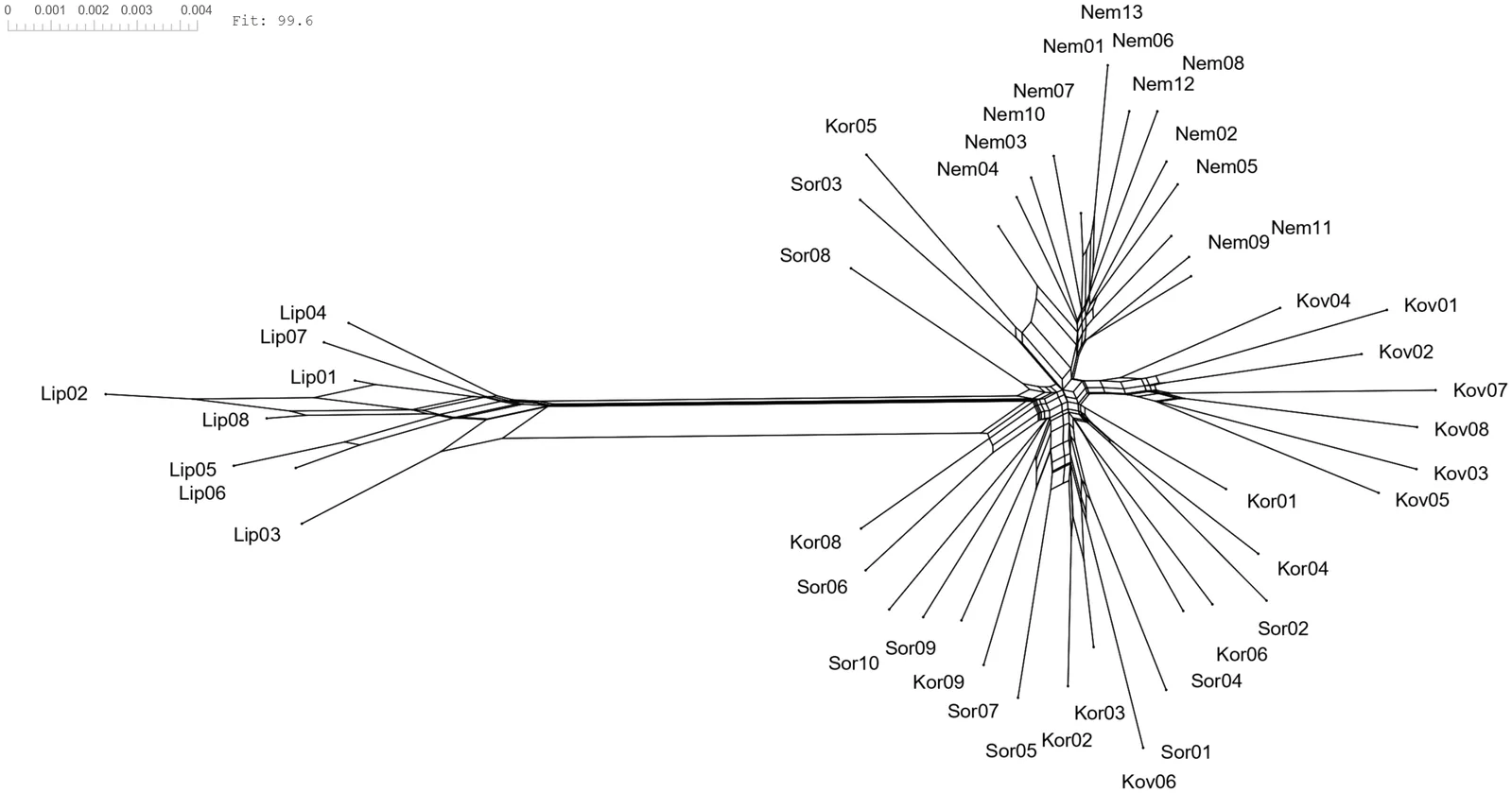
NeighborNet network based on the final filtered RADseq SNP dataset. The network was constructed in SplitsTree from the thinned dataset containing one randomly selected SNP per RAD locus.

### 3.4 Pairwise F_ST_

Pairwise F_ST_ estimates confirmed the strong differentiation of Lipce and the close relationship between Tapolca and Lesencetomaj (**Figure 6**). The highest values (**Table 2**) involved Lipce: Nemeshany–Lipce (weighted F_ST_ = 0.2617; mean site F_ST_ = 0.159, 95% CI = 0.155–0.162), Köveskál–Lipce (weighted F_ST_ = 0.2387; mean site F_ST_ = 0.137, 95% CI = 0.134–0.140), Tapolca–Lipce (weighted F_ST_ = 0.2200; mean site F_ST_ = 0.124, 95% CI = 0.121–0.126) and Lesencetomaj–Lipce (weighted F_ST_ = 0.2122; mean site FST = 0.115, 95% CI = 0.113–0.118). Differentiation among Hungarian populations was lower, with the smallest estimate observed between Tapolca and Lesencetomaj (weighted F_ST_ = 0.0015; mean site F_ST_ = -0.00144, 95% CI = -0.00240 to -0.000486). In contrast, Köveskál was weakly but consistently differentiated from both Lesence localities (mean site F_ST_ = 0.0172, 95% CI = 0.0158–0.0185; mean site F_ST_ = 0.0166, 95% CI = 0.0154–0.0178), whereas Nemeshany showed stronger differentiation from all other Hungarian populations. Bootstrap resampling of site-wise F_ST_ estimates indicated narrow confidence intervals for all pairwise comparisons, showing that the relative differentiation patterns were robust to marker resampling (**Table 2**). As a phenetic summary of relative F_ST_ differentiation, UPGMA dendrogram based on the pairwise F_ST_ matrix grouped Tapolca and Lesencetomaj together, placed Köveskál close to this pair, and separated Nemeshany from the other Hungarian populations.

**Figure 6:**
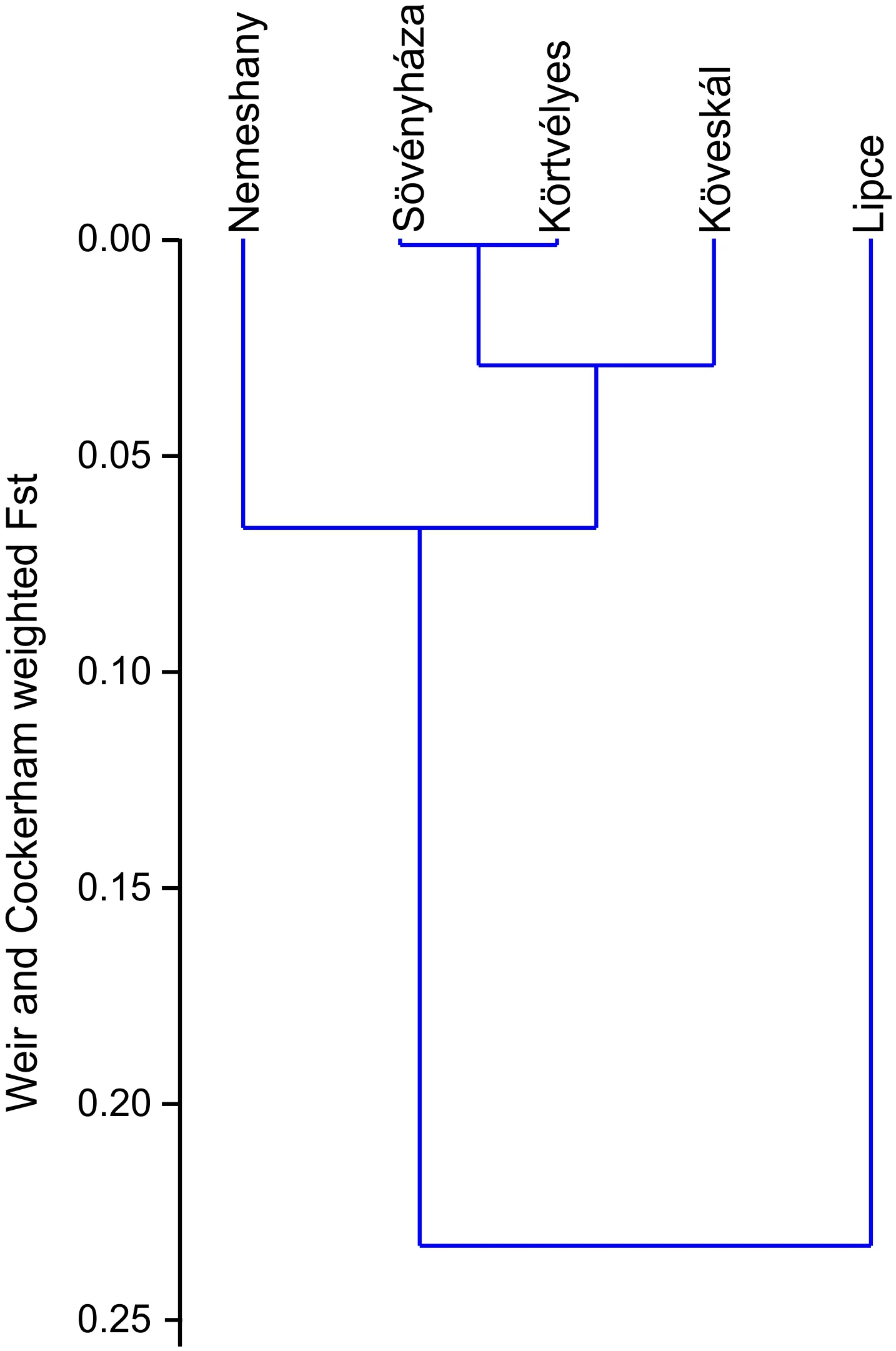
Pairwise F_ST_ clustering among populations. UPGMA dendrogram based on weighted Weir and Cockerham F_ST_ estimates calculated from the filtered one-SNP-per-RAD-locus RADseq dataset. The dendrogram is a phenetic summary of relative differentiation.

**Table 2:** Pairwise Weir and Cockerham F_ST_ estimates among sampled *Primula farinosa* populations.

Comparison	N_SNPs_	Weighted F_ST_	Mean site-wise F_ST_	95% bootstrap CI
Tapolca *vs.* Köveskál	17,973	0.029	0.017	0.016–0.019
Tapolca *vs.* Lipce	20,378	0.220	0.124	0.121–0.126
Tapolca *vs.* Nemeshany	18,045	0.061	0.043	0.041–0.044
Tapolca *vs.* Lesencetomaj	19,437	0.001	-0.001	-0.002–0.000
Köveskál *vs.* Lipce	19,264	0.239	0.137	0.134–0.140
Köveskál *vs.* Nemeshany	17,021	0.073	0.049	0.047–0.050
Köveskál *vs.* Lesencetomaj	19,025	0.030	0.017	0.015–0.018
Lipce *vs.* Nemeshany	18,573	0.262	0.159	0.155–0.162
Lipce *vs.* Lesencetomaj	21,508	0.212	0.115	0.113–0.118
Nemeshany *vs.* Lesencetomaj	19,143	0.066	0.042	0.041–0.044

Weighted F_ST_ values were obtained from vcftools. Mean site-wise F_ST_ values and 95% bootstrap confidence intervals were calculated by non-parametric resampling across SNPs/loci using site-wise F_ST_ estimates. Confidence interval (CI) values represent the 2.5% and 97.5% percentiles of the bootstrap distribution.

### 3.5 Allelic richness and private alleles

Rarefied allelic richness and private-allele analyses revealed clear differences in population-level allelic diversity (**Table 3**). Mean rarefied allelic richness was highest in Lipce (1.321; 95% CI: 1.32–1.33) and Lesence (1.302; 95% CI: 1.30–1.31), intermediate in Balaton (1.266; 95% CI: 1.26–1.27) and Köveskál (1.248; 95% CI: 1.24–1.25), and lowest in Nemeshany (1.178; 95% CI: 1.17–1.18). These results indicate that Lesence retains comparatively high allelic diversity among the Hungarian populations, while Köveskál and especially Nemeshany are genetically depauperate.

**Table 3:** Rarefied allelic richness (with 95% CI values) and private allelic variation by population or regional grouping.

Population	Mean rarified allelic richness (95% CI)	Private alleles total no.	Private alleles no. loci with	Private alleles mean per locus (95% CI)
Lipce	1.321 (1.32–1.33)	18,775	6,519	1.033 (1.005–1.062)
Balaton Region	1.266 (1.26–1.27)	76,254	19,564	2.924 (2.905–2.964)
Lesence	1.302 (1.30–1.31)	14,449	8,304	0.795 (0.778–0.812)
Köveskál	1.248 (1.24–1.25)	3,084	1,985	0.170 (0.162–0.178)
Nemeshany	1.178 (1.17–1.18)	2,714	1,362	0.149 (0.140–0.159)

Private-allele summaries showed a similar but sampling-sensitive pattern. Nemeshany and Köveskál had few private alleles and low mean numbers of private alleles per locus (0.149 and 0.170, respectively). Lipce and Lesence showed higher rarefied allelic richness and more private variation than Nemeshany and Köveskál. At the regional level, Balaton had the highest total number of private alleles and the largest number of loci carrying private alleles; however, raw total private-allele counts were interpreted cautiously because they are affected by unequal sample size and missing-data structure.

To account for the evident dependence of private allele numbers on sample size, we calculated mean private alleles per population and regional groups. In this respect, Lipce showed the highest value (1.033; 95% CI: 1.005–1.062), followed by Lesence (0.795; 95% CI: 0.778–0.812). Köveskál and Nemeshany had substantially lower values, with overlapping or nearly overlapping confidence intervals (Köveskál: 0.170; 95% CI: 0.162–0.178; Nemeshany: 0.149; 95% CI: 0.140–0.159). Thus, private variation was concentrated mainly in Lipce and Lesence, whereas the two more depauperate Hungarian populations carried relatively little private variation per locus. At the regional level, the pooled Balaton Region group showed high private variation, but this comparison should be interpreted cautiously because the Balaton group contained substantially more individuals than Lipce (**Table 3**).

### 3.6 Historical effective population size trajectories

Stairway Plot analyses on independently generated population-specific folded SFSs suggested population-specific differences in historical effective population-size trajectories (**Figure 7**). Lesence showed the highest inferred Ne over much of the reconstructed time range, followed by a marked decline towards the present. Köveskál showed a broadly similar but less pronounced pattern, whereas Nemeshany displayed a strong reduction from formerly higher inferred Ne. Lipnice showed a comparatively lower and more stable trajectory. To assess the sensitivity of temporal interpretation to generation time, the analysis was repeated using a five-year generation time (**Supplementary Figure 1**). This sensitivity analysis recovered the same broad population-specific patterns, including the comparatively high inferred Ne of Lesence, the relative stability of Lipce, and the pronounced decline in Nemeshany, but shifted the inferred timing of demographic changes further into the past and altered the absolute Ne estimates. These differences confirm that the demographic reconstructions are sensitive to scaling assumptions. Nevertheless, all reconstructions showed reduced temporal resolution in older parts of the curves, approximately beyond the Late Glacial interval. These results should therefore be interpreted as broad, population-specific demographic signals rather than precise estimates of the timing or magnitude of historical events. The apparent loss of signal beyond approximately 15 kya was treated as a limitation of the information contained in the folded RADseq site-frequency spectra, not as evidence for a specific demographic event. We therefore interpret these reconstructions as exploratory and do not use them to infer the exact timing of population origin or isolation.

**Figure 7:**
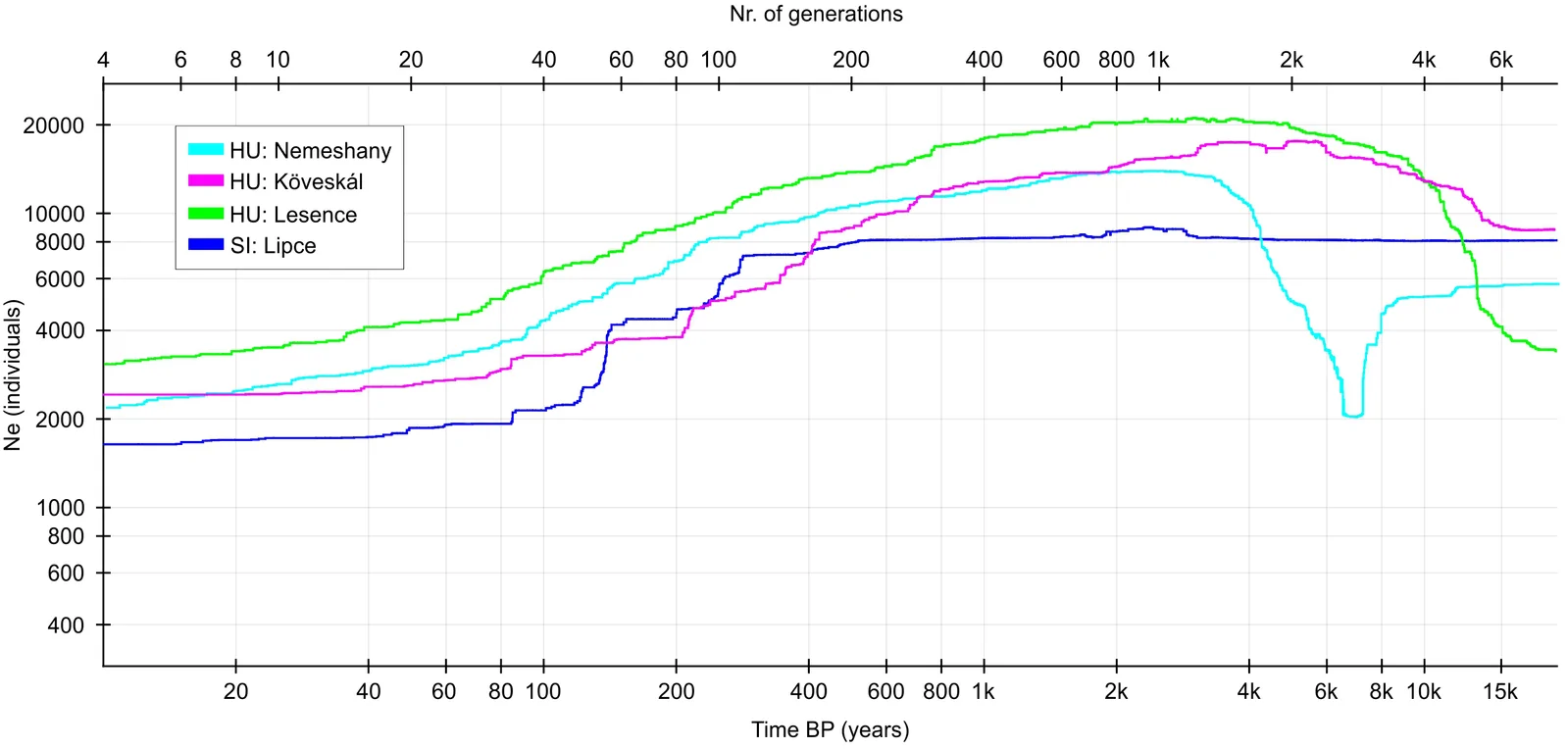
Historical effective population size trajectories. Stairway Plot 2 reconstructions based on independently generated population-specific folded site-frequency spectra. Curves should be interpreted as broad recent-to-mid-Holocene demographic signals rather than precise reconstructions of effective population-size change because temporal resolution declines in older intervals and the analysis is based on reduced-representation genomic data.

## 4 Discussion

This study shows that the low-elevation populations of *Primula farinosa* sampled in Hungary and Slovenia contain geographically structured genomic diversity. The combined evidence from SNP-based tree inference, PCA, ancestry clustering, allelic richness and private allelic variation points to a hierarchically structured and regionally distinctive genomic pattern. Our analyses provide strong evidence for the regional-level population-genomic differentiation between the sampled *P. farinosa* populations. This result has direct conservation relevance because the Balaton Region populations are not genetically interchangeable with Lipce, and several Hungarian populations are also differentiated from one another. However, the results do not allow the exact timing of this differentiation to be resolved. In particular, the present RADseq dataset cannot distinguish unequivocally among uninterrupted local glacial persistence, older regional persistence with habitat shifts, remnants of an early-Holocene distribution, or more recent founder and drift processes. We therefore interpret the Balaton Region populations as a conservation-relevant component of regional genomic diversity, while treating long-term relict persistence as a plausible but not directly demonstrated historical scenario.

### 4.1 Evolutionary history of the Hungarian low-elevation populations

The Hungarian populations are best interpreted as components of the local Balaton Region gene pool of *P. farinosa*, whose present genomic structure has been shaped by geographic isolation, restricted gene flow, drift and relatively recent local demographic processes. Population structure analyses (**Figures 2**–**5**) place all Hungarian samples apart from Lipce, indicating that the Balaton Region populations are genetically differentiated from the Slovenian comparative population. This first-order split is important because Lipce is also a low-elevation population close to the Alps; therefore, the Hungarian populations are not simply genetically similar low-elevation satellites of the Slovenian Alpine-margin population. The Hungarian stands instead appear to preserve a separate regional genomic signature.

Within Hungary, the Lesence Basin populations, Körtvélyes and Sövényháza, are nearly indistinguishable in all structure analyses (**Figures 2**–**5**) and show almost no pairwise differentiation (**Figure 6**). Their treatment as a single biological population is therefore justified for broad population-genomic interpretation, even though management should still consider local habitat conditions separately. Lesence also has one of the highest rarefied allelic-richness values in the dataset, suggesting that it has avoided stronger recent bottlenecks affecting the smaller and more isolated Hungarian stands. This contrast between field observations of repeated apparent disappearance (Salamon-Albert and Morschhauser, 2003) and genomic diversity suggests that demographic census size and retained genomic variation may be partly decoupled. Lesence may have experienced recent demographic fluctuation or local disappearance–recolonization dynamics while still retaining a relatively diverse regional gene pool. By contrast, Nemeshany and Köveskál form recognizable genetic clusters (**Figures 2**–**5**) and show reduced allelic richness (**Table 3**). Together with the demographic reconstructions, which indicate declines in inferred effective population size in these populations (**Figure 7**; **Supplementary Figure 1**), this pattern is most consistent with demographic bottlenecks followed by strong genetic drift in small, isolated stands. This interpretation is especially plausible for Nemeshany, which combines the lowest rarefied allelic richness with a pronounced reduction in inferred Ne. Köveskál shows a less extreme but broadly similar pattern.

The position of Lesence within the Hungarian clade is especially relevant, but should be interpreted cautiously. When rooted with Lipce as the external comparative population, the SNP-based tree (**Figure 2**) placed Lesence (Körtvélyes and Sövényháza) as sister to the Nemeshany and Köveskál group within the Hungarian samples. This topology indicates that Lesence occupies a distinct position in the sampled regional genomic structure, whereas Nemeshany and Köveskál form a more closely related pair that have subsequently accumulated drift and lost allelic variation. While this should not be interpreted as direct evidence that Lesence gave rise to the other Hungarian populations, because a SNP tree is not an explicit demographic model, the interpretation is reinforced by the allelic-richness pattern. The allelic-richness pattern is nevertheless consistent with Lesence having experienced less severe genetic erosion than Nemeshany and Köveskál: Lesence was almost as rich as Lipce, whereas Nemeshany was the most depauperate population and Köveskál was only slightly richer (**Table 3**). Thus, Lesence may represent a relatively less eroded component of the Balaton Region gene pool, although alternative explanations, including differences in effective population size, drift, bottleneck severity or historical connectivity, cannot be excluded.

It is also notable that one Köveskál individual (#06) consistently groups with individuals from Lesence (**Figures 2**–**4**). That individual was sampled in the western part of the Köveskál population and consistently shows affinity to Lesence in independent analyses suggesting it is not an analytical noise. Although alternative scenarios, including natural gene flow, shared ancestry, incomplete lineage sorting, local genetic heterogeneity, or sample-label/genotyping issues, cannot be fully excluded, the documented sowing of Lesence propagules for conservation reasons at Köveskál provides an independent management context that makes anthropogenic introduction a plausible hypothesis for this pattern. Indeed, records from the Balaton-felvidéki National Park Directorate indicate repeated sowing of Lesence propagules at Köveskál since 2006, when the habitat began to suffer from drying. Despite these repeated introductions, the Lesence-like genomic assignment was detected in only a single individual from the western part of the population. This may indicate limited success of the sowing efforts and/or the persistence of the prevailing local genotype, although confirmation would require genetic analysis of the actual material used for sowing or of individuals known to derive from the introduced propagules. Given the short-term persistent seed bank reported for the species (Lindborg and Ehrlén, 2002), the limited genomic signal of sowing may also reflect restricted recruitment from introduced seeds, although this was not directly tested here.

Thus, the Hungarian populations combine evidence of regional distinctiveness, local demographic erosion and recent management-mediated genetic input, emphasizing that their present genomic structure likely reflects multiple historical and contemporary processes rather than a single recent event.

### 4.2 Relationship to the interglacial-expansion model in *Primula farinosa*

At the species-wide scale*, P. farinosa* has already been presented as a strong example of the interglacial-expansion model for cold-adapted plants (Theodoridis et al., 2017). This contrasts with many European high-mountain plants, for which molecular syntheses and comparative phylogeographic studies often point to survival in peripheral, nunatak or lowland refugia followed by postglacial range rearrangement (Schönswetter et al., 2005; Holderegger and Thiel-Egenter, 2009; Stewart et al., 2010). This scenario is biologically plausible because *P. farinosa* is not a strict high-alpine specialist: it is a moisture-demanding, weakly competitive plant of open calcareous habitats. During cold stages, many Central European lowland landscapes were characterized by cold steppe or steppe-tundra vegetation mosaics under strongly continental climatic conditions. Such landscapes may have contained extensive open habitats, but they were not necessarily suitable for a fen specialist whose persistence depends on continuously wet, calcareous and competition-limited microhabitats (Birks, 2008; Fletcher et al., 2010; Theodoridis et al., 2013, Theodoridis et al., 2017). Therefore, potential refugia for *P. farinosa* should be sought primarily in locally buffered wet habitats, such as calcareous fens, spring-fed wetlands, lake margins or other groundwater-influenced open sites, rather than in generalized steppe landscapes.

Our results add a finer regional layer to that picture. Pairwise F_ST_ provides the most direct measure of the extent of genetic differentiation among the sampled populations. The narrow bootstrap confidence intervals of our analysis indicate that the observed hierarchy of genetic differentiation is not driven by stochastic marker sampling. Viewed against the broad comparative patterns summarized by Gamba and Muchhala (2020)—where outcrossing and temperate herb species generally show lower population differentiation, whereas small-insect pollination is associated with higher differentiation—the Lipce–Balaton Region comparisons indicate substantial regional differentiation within a conspecific, predominantly outcrossing temperate herb, with weighted F_ST_ values of 0.2122–0.2617 (**Figure 6**). These values are far above the almost complete lack of differentiation between Körtvélyes and Sövényháza (weighted F_ST_ = 0.0015), and they are also higher than the differentiation among most Hungarian populations. The contrast is informative. Very low F_ST_ within Lesence is consistent with recent connectivity or a shared local gene pool, whereas high F_ST_ between Lipce and the Balaton Region populations is compatible with prolonged restriction of gene flow (including drift, founder effects, bottlenecks and long-term small effective population size) and/or independent regional demographic histories.

While several microevolutionary processes can contribute to the observed pattern of strong regional differentiation, these results do not contradict the interglacial-expansion model. Rather, they suggest that the present-day Balaton Region occurrences are unlikely to be the product of very recent connectivity with the Alpine-margin low-elevation population. If such *P. farinosa* had spread widely across the region during the Holocene and only very recently contracted into scattered fen remnants, we would expect weaker differentiation between the Slovenian and Hungarian low-elevation populations, and probably more homogeneous ancestry among the Hungarian populations. Instead, the strong Slovenian–Hungarian split and the internal structure among Hungarian stands imply that low-elevation persistence and fragmentation operated over a longer or more complex timescale. Comparable cases in European wetland and subalpine plants show that strongly separated lineages may persist in geographically scattered refugial or peripheral habitats and can remain historically informative even when demographic timing is uncertain (Lienert et al., 2002; Urbaniak and Kwiatkowski, 2023). Thus, although the present results do not prove uninterrupted *in situ* survival at each sampled Hungarian site since the Late Glacial, they strongly support the conservation-genetic conclusion that the Balaton Region populations represent an isolated and conservation-relevant regional component of *P. farinosa* diversity. Their possible relictual origin remains a plausible historical interpretation, but it should be treated as a hypothesis requiring further testing rather than as a demonstrated result of the present RADseq dataset.

This interpretation fits the ecology of *P. farinosa* as a moisture-demanding, weakly competitive species of open calcareous habitats. In the Carpathian Basin, dry LGM vegetation and later Holocene forest expansion would both have constrained the species outside specialized wet calcareous microhabitats (Magyari et al., 2010, Magyari et al., 2014; Feurdean et al., 2014). Central European relict-rich fens provide a more relevant context than comparisons with strict high-mountain species. Hájek et al. (2011) showed that several fen plants and terrestrial snails display Holocene-scale relict distribution patterns, while Dítě et al. (2018) argued that many Western Carpathian glacial-relict candidates are linked to habitats that were regionally more widespread under colder or more open past landscapes, including peatlands and mineral-rich fens. Thus, any refugial interpretation of the Balaton Region populations should be understood in terms of locally buffered wetland refugia, such as calcareous fens, spring-fed wetlands or lake-margin habitats, rather than generalized high-mountain or steppe-refugial models (Stewart et al., 2010; Hájek et al., 2006, Hájek et al., 2011). The Balaton populations therefore make biogeographic sense as isolated low-elevation fen populations maintained by local edaphic and hydrological conditions, although their exact historical origin remains unresolved. Seen against this background, the Hungarian populations of *P. farinosa* fit a recognized Eurasian pattern: southern or Central-Eastern European low-elevation populations of cold-adapted taxa survive only where local habitat conditions buffer them from the surrounding warmer, more competitive landscape.

### 4.3 Demographic reconstruction and unresolved timing of isolation

One aim of this study was to test whether demographic reconstruction could provide an independent evidence for the relict status of the Balaton Region populations. However, the Stairway Plot analyses did not allow a reliable reconstruction of the timing of population origin or isolation. The generation-time sensitivity analysis further supports a cautious interpretation of the Stairway Plot results: the 2.5-year and 5-year analyses produced broadly similar relative patterns among populations, but differed in the calendar timing of inferred changes and in absolute Ne estimates. Although the population-specific curves suggest demographic heterogeneity among the sampled populations, the older segments of the trajectories, particularly beyond approximately 15 kya, were weakly resolved, probably reflecting the limited information content of folded site-frequency spectra derived from RADseq data. Demographic reconstruction methods based on linkage disequilibrium could provide a powerful alternative to SFS-based inference, but they require reliable genomic coordinates of markers. Because our *de novo* RADseq dataset lacked absolute genomic positions, such analyses were not feasible in the present study.

Therefore, these analyses should be interpreted only as broad indicators of population-specific demographic differences, not as precise reconstructions of Late Glacial or Holocene history. The present data cannot distinguish whether the Hungarian populations represent continuous local survivors, older regional relicts that shifted among suitable fen habitats, or remnants of an early Holocene lowland distribution that became isolated before modern habitat fragmentation. Nevertheless, when considered together with the strong regional differentiation, the distinct Balaton Region genomic signature and the reduced allelic richness of some Hungarian populations, the demographic results are compatible with potential long-term isolation, while not providing direct evidence for it.

### 4.4 Comparison with other low-elevation relicts and fragmented wetland specialists

The general phylogeographic pattern observed here resembles several other European cases in which lowland, peripheral or isolated populations of cold-adapted plants retain strong regional structure but differ in genetic diversity depending on isolation history and local demographic stability (Ægisdóttir et al., 2009; Reisch and Rosbakh, 2021). Comparisons with wet-habitat species at southern or fragmented parts of their ranges provide a more appropriate context than analogies with strict high-mountain specialists. These comparisons are relevant because, like *P. farinosa* in the Balaton Region, such species often persist in isolated wetland habitats where local hydrology, openness and low nutrient availability maintain suitable conditions despite an otherwise unsuitable regional landscape.

In *Saxifraga hirculus* L., a circumpolar species of wet habitats, European populations were strongly differentiated and had lower haplotype diversity than Alaskan populations, yet, chloroplast data did not identify clear European migration routes or refugia (Oliver et al., 2006). This is a useful cautionary parallel: strong geographic genetic structure can be well established even when the location and age of historical refugia remain unresolved. The cloudberry, *Rubus chamaemorus* L., provides another wet-habitat comparison. Phylogeographic work combined with pollen evidence showed that this peatland species could persist through dry glacial climates in restricted regions (Ehrich et al., 2008), while later analyses found strong differentiation between Czech and Norwegian populations and unique regional allele compositions in marginal populations (Leišová-Svobodová et al., 2018). In *Ligularia sibirica* Cass., isolated Central European wetland populations retained substantial within-population diversity and showed geographically structured differentiation, demonstrating that remnant wetland populations need not share a single simple diversity signature (Šmídová et al., 2011). For *Swertia perennis* L., another cold-adapted wetland plant with disjunct European lowland and montane occurrences, Lienert et al. (2002) reported that fen size and isolation affected isozyme variability. A later phylogeographic study of the species recovered several major Central European lineages and interpreted some north-eastern Polish and German populations as relics of formerly wider periglacial genetic groups (Urbaniak and Kwiatkowski, 2023).

Species-specific and congeneric studies provide useful context. Reisch et al. (2005) found significant altitudinal genetic differentiation among ten northern Alpine *P. farinosa* populations, with higher-altitude populations tending to retain greater within-population diversity; the authors attributed this pattern to phenological differences and vegetation-mediated restrictions on gene flow. A RAPD survey of four Lithuanian *P. farinosa* populations likewise found each sampled population genetically distinct (Augėnaitė et al., 2010). In north-eastern Switzerland, habitat fragmentation in *P. farinosa* itself reduced population size and altered performance-related ecological conditions in fen fragments (Lienert and Fischer, 2003). These studies make the present allelic-richness pattern more interpretable: the low diversity of Nemeshany and Köveskál is not unexpected for small, isolated fen populations, especially in an outcrossing and heterostylous species where demographic decline can quickly interact with mate limitation, pollinator limitation and genetic drift. More generally, they show that habitat configuration, isolation and restricted gene flow can be associated with substantial population structure in wetland-associated *Primula* populations, without necessarily providing a direct estimate of the age of that structure.

### 4.5 Allelic richness and private variation: conservation implications

Rarefied allelic richness is central to the conservation interpretation because it allows comparison among populations with different sample sizes. Lipce and Lesence have the highest values, suggesting that these populations retain more standing variation and may have experienced less severe recent genetic erosion. In contrast, Nemeshany and Köveskál showed lower allelic richness, with Nemeshany showing the lowest rarefied allelic richness and low private-allele values. However, given the modest population-level sample sizes, small differences among populations should not be overinterpreted. Low diversity can indicate recent founder events or bottlenecks, but in rear-edge and relict populations it can also reflect long-term persistence at small effective population size (Hampe and Petit, 2005). The elevated differentiation among several Balaton Region populations is compatible with this latter possibility, but, admittedly, it is not diagnostic of it, because similar patterns may also result from recent drift, founder events, bottlenecks or restricted gene flow. Broad comparative work on European plants also shows that isolated peripheral populations may combine reduced within-population diversity with stronger among-population differentiation, so low allelic richness should not be equated automatically with low historical or conservation value (Reisch and Rosbakh, 2021). Such populations may still contain unique allele combinations, locally adapted genotypes or population-specific components of genetic variation, even if their genome-wide diversity is reduced.

The private-allele results should be read cautiously. At the regional scale, Balaton has many private alleles and many loci carrying private alleles, but this total is affected by the larger number of samples pooled into the regional group. This limitation is especially relevant for the regional comparison, where the pooled Balaton Region sample contains substantially more individuals than Lipce. For this reason, we do not interpret raw private-allele totals as directly comparable estimates of regional uniqueness. Instead, we emphasize rarefied allelic richness as the main standardized diversity measure and use mean private alleles per locus, with bootstrap confidence intervals, as a supplementary description of private variation under the observed sampling design. At the population level, this analysis showed higher mean private alleles per locus in Lipce and Lesence than in Köveskál and Nemeshany, whereas raw regional private-allele totals were retained only as descriptive summaries. The stronger conservation point is therefore not the absolute number of private alleles, but the concordant evidence that the Balaton Region populations are genomically distinctive from Lipce, and several Hungarian populations are also distinguishable from one another (**Table 3**). For conservation, this argues against replacing one population with another or treating all low-elevation populations as a single exchangeable unit. Lesence may be valuable as a relatively allele-rich Hungarian reservoir, whereas Nemeshany and Köveskál may be valuable because they represent differentiated and potentially historically isolated fragments of the Balaton Region gene pool. Both types of value matter, because conservation based only on high diversity can overlook historically distinctive but genetically eroded populations.

### 4.6 Broader significance of southern low-elevation refugia

The Balaton Region populations belong to a broader biogeographic phenomenon: low-elevation occurrences of cold-adapted or high-mountain plants at southern latitudes can persist where local habitat conditions reproduce some of the ecological circumstances otherwise associated with colder regions or higher elevations. In Europe, such habitats include calcareous fens, spring systems, wet meadows, river gravels, cliffs and other open, competition-limited refugia (Birks, 2008; Hájek et al., 2006, Hájek et al., 2011; Horsák et al., 2015; Holderegger and Thiel-Egenter, 2009). Similar logic also applies elsewhere in temperate regions. Circumpolar and East Asian arctic-alpine studies show that disjunct mountain or peripheral populations can retain complex histories of isolation, connectivity and refugial persistence (Eidesen et al., 2013; Ikeda et al., 2016), while Lake Superior shoreline populations illustrate how cool, wet, exposed microhabitats can maintain southern disjunct arctic-alpine plant assemblages far from their main ranges (Hillman and Nielsen, 2024). These populations are often small, fragmented and genetically idiosyncratic, but that is precisely why they are biogeographically valuable.

In this wider context, Hungarian *P. farinosa* should not be viewed as a botanical curiosity sitting awkwardly outside the “real” mountain range of the species. Rather, its presence is compatible with a relict-refugial system in which low-elevation wetland habitats can preserve a genetic structure long after regional climate and vegetation have changed. The conservation implication is clear: maintaining multiple isolated populations is more important than simply protecting the largest or most allele-rich site. Lesence, Köveskál and Nemeshany each contribute differently to the regional genomic pattern. Lesence retains higher allelic richness, Nemeshany and Köveskál preserve differentiated but genetically eroded components, and the Balaton Region as a whole is strongly distinct from the Slovenian Alpine-margin low-elevation population. In light of the potential rear-edge nature of these populations (see Hampe and Petit, 2005), future conservation should therefore combine genetic management with practical conservation. In practical terms, this should be combined with continued maintenance of suitable habitat conditions, especially hydrology, open fen vegetation and low nutrient status. Additional non-genomic monitoring, including population size, recruitment and floral morph ratios in this distylous species, would be valuable future management considerations, although these parameters were not directly tested in the present study.

Taken together, our results indicate that the Balaton Region populations of *P. farinosa* represent a distinct and conservation-relevant component of regional genomic diversity. The strongest conclusion of this study is that these populations contain geographically structured and non-interchangeable genomic variation: they are strongly differentiated from Lipce, show internal structure within Hungary, and differ in the extent of allelic erosion. This conservation-genetic conclusion does not depend on demonstrating uninterrupted Glacial persistence. Although the present data do not resolve the exact timing of their isolation, they argue against treating them as recent, interchangeable remnants of a single homogeneous low-elevation population. Their conservation should therefore maintain the distinctiveness of individual populations while also addressing the local habitat processes that determine their persistence.

## Data Availability

The datasets presented in this study can be found in online repositories. The names of the repository/repositories and accession number(s) can be found below: https://www.ncbi.nlm.nih.gov/, PRJNA1498655.

## Supplementary material

Supplementary Figure 1 Bootstrap uncertainty of Stairway Plot demographic reconstructions and generation time sensitivity results for the four analyzed *Primula farinosa* populations. Panel **(A)** shows reconstructions scaled with a generation time of 2.5 years, whereas panel **(B)** shows the corresponding sensitivity analysis using a generation time of 5 years. Red lines indicate median inferred Ne trajectories, and grey lines show bootstrap uncertainty intervals.

## References

[B2] Alexander D. H. Novembre J. Lange K. (2009). Fast model-based estimation of ancestry in unrelated individuals. Genome Res. 19, 1655–1666. doi: 10.1101/gr.094052.109 19648217 PMC2752134

[B3] Anisimova M. Gascuel O. (2006). Approximate likelihood-ratio test for branches: a fast, accurate, and powerful alternative. Syst. Biol. 55, 539–552. doi: 10.1080/10635150600755453 16785212

[B4] Augėnaitė V. Žvingila D. Meškauskaitė E. Naujalis J. R. (2010). Raktažolės pelenėlės (*Primula farinosa* L.) genetiniai tyrimai Lietuvoje [Genetic studies of *Primula farinosa* L. in Lithuania. Vytauto Didžiojo universiteto Botanikos sodo raštai 14, 8–14.

[B5] Baird N. A. Etter P. D. Atwood T. S. Currey M. C. Shiver A. L. Lewis Z. A. . (2008). Rapid SNP discovery and genetic mapping using sequenced RAD markers. PloS One 3, e3376. doi: 10.1371/journal.pone.0003376 18852878 PMC2557064

[B6] Birks H. H. (2008). The Late-Quaternary history of arctic and alpine plants. Plant Ecol. Divers. 1, 135–146. doi: 10.1080/1755087080232865237339054

[B7] Borbás V. (1900). A Balaton Tavának És Partmellékének Növényföldrajza És Edényes Növényzete [Phytogeography and Vascular Flora of Lake Balaton and its Surrounding Area] (Budapest: Hornyánszky Viktor Csász. és Kir. Udvari Könyvnyomdája).

[B8] Borhidi A. Kevey B. Lendvai G. (2012). Plant Communities of Hungary (Budapest: Akadémiai Kiadó).

[B9] Bryant D. Moulton V. (2004). Neighbor-Net: an agglomerative method for the construction of phylogenetic networks. Mol. Biol. Evol. 21, 255–265. doi: 10.1093/molbev/msh018 14660700

[B10] Catchen J. Hohenlohe P. A. Bassham S. Amores A. Cresko W. A. (2013). Stacks: an analysis tool set for population genomics. Mol. Ecol. 22, 3124–3140. doi: 10.1111/mec.12354 23701397 PMC3936987

[B11] Chang C. C. Chow C. C. Tellier L. C. A. M. Vattikuti S. Purcell S. M. Lee J. J. (2015). Second-generation PLINK: rising to the challenge of larger and richer datasets. GigaScience 4, 7. doi: 10.1186/s13742-015-0047-8 25722852 PMC4342193

[B12] Chen S. Zhou Y. Chen Y. Gu J. (2018). fastp: an ultra-fast all-in-one FASTQ preprocessor. Bioinformatics 34, i884–i890. doi: 10.1093/bioinformatics/bty560 30423086 PMC6129281

[B13] Crawford R. M. M. (2008). Cold climate plants in a warmer world. Plant Ecol. Divers. 1, 285–297. doi: 10.1080/1755087080240733237339054

[B14] Dalén L. Nyström V. Valdiosera C. Germonpré M. Sablin M. Turner E. . (2007). Ancient DNA reveals lack of postglacial habitat tracking in the arctic fox. Proc. Natl. Acad. Sci. U.S.A. 104, 6726–6731. doi: 10.1073/pnas.0701341104 17420452 PMC1871853

[B15] Danecek P. Auton A. Abecasis G. Albers C. A. Banks E. DePristo M. A. . (2011). The variant call format and VCFtools. Bioinformatics 27, 2156–2158. doi: 10.1093/bioinformatics/btr330 21653522 PMC3137218

[B16] de Vos J. M. Hughes C. E. Schneeweiss G. M. Moore B. R. Conti E. (2014). Heterostyly accelerates diversification via reduced extinction in primroses. Proc. R. Soc B. 281, 20140075. doi: 10.1098/rspb.2014.0075 24759859 PMC4043087

[B17] Dítě D. Hájek M. Svitková I. Košuthová A. Šoltés R. Kliment J. (2018). Glacial-relict symptoms in the Western Carpathian flora. Folia Geobot 53, 277–300. doi: 10.1007/s12224-018-9321-830311153

[B1] Ægisdóttir H. H. Kuss P. Stöcklin J. (2009). Isolated populations of a rare alpine plant show high genetic diversity and considerable population differentiation. Ann. Bot. 104, 1313–1322. doi: 10.1093/aob/mcp242 19797423 PMC2778390

[B18] Ehrich D. Alsos I. G. Brochmann C. (2008). Where did the northern peatland species survive the dry glacials: cloudberry (*Rubus chamaemorus*) as an example. J. Biogeogr. 35, 801–814. doi: 10.1111/j.1365-2699.2007.01864.x40046247

[B19] Eidesen P. B. Ehrich D. Bakkestuen V. Alsos I. G. Gilg O. Taberlet P. . (2013). Genetic roadmap of the Arctic: plant dispersal highways, traffic barriers and capitals of diversity. New Phytol. 200, 898–910. doi: 10.1111/nph.12412 23869846

[B20] El Mousadik A. Petit R. J. (1996). High level of genetic differentiation for allelic richness among populations of the argan tree [*Argania spinosa* (L.) Skeels] endemic to Morocco. Theor. Appl. Genet. 92, 832–839. doi: 10.1007/BF00221895 24166548

[B21] Espíndola A. Pellissier L. Maiorano L. Hordijk W. Guisan A. Alvarez N. (2012). Predicting present and future intra-specific genetic structure through niche hindcasting across 24 millennia. Ecol. Lett. 15, 649–657. doi: 10.1111/j.1461-0248.2012.01779.x 22515791

[B22] Feurdean A. Perşoiu A. Tanţău I. Stevens T. Magyari E. K. Onac B. P. . (2014). Climate variability and associated vegetation response throughout Central and Eastern Europe (CEE) between 60 and 8 ka. Quat. Sci. Rev. 106, 206–224. doi: 10.1016/j.quascirev.2014.06.00342574925

[B23] Fletcher W. Sánchez Goñi M. F. Allen J. M. R. Cheddadi R. Combourieu-Nebout N. Huntley B. . (2010). Millennial-scale variability during the last glacial in vegetation records from Europe. Quat. Sci. Rev. 29, 2839–2864. doi: 10.1016/j.quascirev.2009.11.01542574925

[B24] Gamba D. Muchhala N. (2020). Global patterns of population genetic differentiation in seed plants. Mol. Ecol. 29, 3413–3428. doi: 10.1111/mec.15575 32743850

[B25] Goudet J. (2005). hierfstat, a package for R to compute and test hierarchical F-statistics. Mol. Ecol. Notes 5, 184–186. doi: 10.1111/j.1471-8286.2004.00828.x40046247

[B30] Hájek M. Horsák M. Hájková P. Dítě D. (2006). Habitat diversity of central European fens in relation to environmental gradients and an effort to standardise fen terminology in ecological studies. Perspect. Plant Ecol. Evol. Syst. 8, 97–110. doi: 10.1016/j.ppees.2006.08.00242574925

[B31] Hájek M. Horsák M. Tichý L. Hájková P. Dítě D. Jamrichová E. (2011). Testing a relict distributional pattern of fen plant and terrestrial snail species at the Holocene scale: a null model approach. J. Biogeogr. 38, 742–755. doi: 10.1111/j.1365-2699.2010.02424.x40046247

[B26] Hambler D. J. Dixon J. M. (2003). Primula farinosa L. J. Ecol. 91, 694–705. doi: 10.1046/j.1365-2745.2003.00797.x37945311

[B27] Hammer Ø. Harper D. A. T. Ryan P. D. (2001). PAST: paleontological statistics software package for education and data analysis. Palaeontol Electron. 4, 1–9.

[B28] Hampe A. Petit R. J. (2005). Conserving biodiversity under climate change: the rear edge matters. Ecol. Lett. 8, 461–467. doi: 10.1111/j.1461-0248.2005.00739.x 21352449

[B29] Harrison S. P. Prentice I. C. (2003). Climate and CO2 controls on global vegetation distribution at the last glacial maximum: analysis based on palaeovegetation data, biome modelling and palaeoclimate simulations. Glob. Change Biol. 9, 983–1004. doi: 10.1046/j.1365-2486.2003.00640.x37945311

[B32] Hewitt G. M. (1999). Post-glacial re-colonization of European biota. Biol. J. Linn. Soc 68, 87–112. doi: 10.1111/j.1095-8312.1999.tb01160.x40046247

[B33] Hewitt G. M. (2004). Genetic consequences of climatic oscillations in the Quaternary. Philos. Trans. R. Soc Lond. B. Biol. Sci. 359, 183–195. doi: 10.1098/rstb.2003.1388 15101575 PMC1693318

[B34] Hillman A. Nielsen S. E. (2024). Climate refugia along Lake Superior’s shores: disjunct arctic-alpine plants rely on cool shoreline temperatures but are restricted to highly exposed habitat under climate warming. J. Plant Ecol. 17, rtae050. doi: 10.1093/jpe/rtae050

[B35] Hoang D. T. Chernomor O. von Haeseler A. Minh B. Q. Vinh L. S. (2018). UFBoot2: improving the ultrafast bootstrap approximation. Mol. Biol. Evol. 35, 518–522. doi: 10.1093/molbev/msx281 29077904 PMC5850222

[B36] Holderegger R. Thiel-Egenter C. (2009). A discussion of different types of glacial refugia used in mountain biogeography and phylogeography. J. Biogeogr. 36, 476–480. doi: 10.1111/j.1365-2699.2008.02027.x40046247

[B37] Horsák M. Chytrý M. Hájková P. Hájek M. Danihelka J. Horsáková V. . (2015). European glacial relict snails and plants: environmental context of their modern refugial occurrence in southern Siberia. Boreas 44, 638–657. doi: 10.1111/bor.1213340046247

[B38] Huson D. H. Bryant D. (2024). The SplitsTree App: interactive analysis and visualization using phylogenetic trees and networks. Nat. Methods 21, 1773–1774. doi: 10.1038/s41592-024-02406-3 39223398

[B39] Ikeda H. Sakaguchi S. Yakubov V. Barkalov V. Setoguchi H. (2016). Importance of demographic history for phylogeographic inference on the arctic-alpine plant Phyllodoce caerulea in East Asia. Heredity 116, 232–238. doi: 10.1038/hdy.2015.95 26531250 PMC4806892

[B40] Jombart T. (2008). adegenet: a R package for the multivariate analysis of genetic markers. Bioinformatics 24, 1403–1405. doi: 10.1093/bioinformatics/btn129 18397895

[B41] Kalinowski S. T. (2004). Counting alleles with rarefaction: private alleles and hierarchical sampling designs. Conserv. Genet. 5, 539–553. doi: 10.1023/B:COGE.0000041021.91777.1A

[B42] Kalyaanamoorthy S. Minh B. Q. Wong T. K. F. von Haeseler A. Jermiin L. S. (2017). ModelFinder: fast model selection for accurate phylogenetic estimates. Nat. Methods 14, 587–589. doi: 10.1038/nmeth.4285 28481363 PMC5453245

[B43] Kamvar Z. N. Tabima J. F. Grünwald N. J. (2014). Poppr: an R package for genetic analysis of populations with clonal, partially clonal, and/or sexual reproduction. PeerJ 2, e281. doi: 10.7717/peerj.281 24688859 PMC3961149

[B44] Knaus B. J. Grünwald N. J. (2017). vcfR: a package to manipulate and visualize variant call format data in R. Mol. Ecol. Resour. 17, 44–53. doi: 10.1111/1755-0998.12549 27401132

[B45] Kropf M. Kadereit J. W. Comes H. P. (2003). Differential cycles of range contraction and expansion in European high mountain plants during the Late Quaternary: insights from *Pritzelago alpina* (L.) O. Kuntze (Brassicaceae). Mol. Ecol. 12, 931–949. doi: 10.1046/j.1365-294X.2003.01781.x 12753213

[B46] Leišová-Svobodová L. Phillips J. Martinussen I. Holubec V. (2018). Genetic differentiation of *Rubus chamaemorus* populations in the Czech Republic and Norway after the last glacial period. Ecol. Evol. 8, 5701–5711. doi: 10.1002/ece3.4101 29938086 PMC6010844

[B47] Lewis P. O. (2001). A likelihood approach to estimating phylogeny from discrete morphological character data. Syst. Biol. 50, 913–925. doi: 10.1080/106351501753462876 12116640

[B48] Lienert J. Fischer M. (2003). Habitat fragmentation affects the common wetland specialist *Primula farinosa* in north-east Switzerland. J. Ecol. 91, 587–599. doi: 10.1046/j.1365-2745.2003.00793.x37945311

[B49] Lienert J. Fischer M. Schneller J. J. Diemer M. (2002). Isozyme variability of the wetland specialist *Swertia perennis* (Gentianaceae) in relation to habitat size, isolation, and plant fitness. Am. J. Bot. 89, 801–811. doi: 10.3732/ajb.89.5.801 21665680

[B50] Lindborg R. Ehrlén J. (2002). Evaluating the extinction risk of a perennial herb: demographic data versus historical records. Conserv. Biol. 16, 683–690. doi: 10.1046/j.1523-1739.2002.00509.x37945311

[B51] Liu X. Fu Y. X. (2015). Exploring population size changes using SNP frequency spectra. Nat. Genet. 47, 555–559. doi: 10.1038/ng.3254 25848749 PMC4414822

[B52] Liu X. Fu Y. X. (2020). Stairway plot 2: demographic history inference with folded SNP frequency spectra. Genome Biol. 21, 280. doi: 10.1186/s13059-020-02196-9 33203475 PMC7670622

[B53] Magyari E. K. Chapman J. C. Passmore D. G. Allen J. R. M. Huntley J. P. Huntley B. (2010). Holocene persistence of wooded steppe in the Great Hungarian Plain. J. Biogeogr. 37, 915–935. doi: 10.1111/j.1365-2699.2009.02261.x40046247

[B54] Magyari E. K. Veres D. Wennrich V. Wagner B. Braun M. Jakab G. . (2014). Vegetation and environmental responses to climate forcing during the Last Glacial Maximum and deglaciation in the East Carpathians: attenuated response to maximum cooling and increased biomass burning. Quat. Sci. Rev. 106, 278–298. doi: 10.1016/j.quascirev.2014.09.01542574925

[B55] Minh B. Q. Schmidt H. A. Chernomor O. Schrempf D. Woodhams M. D. von Haeseler A. . (2020). IQ-TREE 2: new models and efficient methods for phylogenetic inference in the genomic era. Mol. Biol. Evol. 37, 1530–1534. doi: 10.1093/molbev/msaa015 32011700 PMC7182206

[B56] Muster C. Berendonk T. U. (2006). Divergence and diversity: lessons from an arctic-alpine distribution (*Pardosa saltuaria* group, Lycosidae). Mol. Ecol. 15, 2921–2933. doi: 10.1111/j.1365-294X.2006.02989.x 16911211

[B57] Oliver C. Hollingsworth P. M. Gornall R. J. (2006). Chloroplast DNA phylogeography of the arctic-montane species *Saxifraga hirculus* (Saxifragaceae). Heredity 96, 222–231. doi: 10.1038/sj.hdy.6800785 16449984

[B58] Paris J. R. Stevens J. R. Catchen J. M. (2017). Lost in parameter space: a road map for Stacks. Methods Ecol. Evol. 8, 1360–1373. doi: 10.1111/2041-210X.1277540046247

[B59] Purcell S. Neale B. Todd-Brown K. Thomas L. Ferreira M. A. R. Bender D. . (2007). PLINK: a tool set for whole-genome association and population-based linkage analyses. Am. J. Hum. Genet. 81, 559–575. doi: 10.1086/519795 17701901 PMC1950838

[B60] Reisch C. Anke A. Röhl M. (2005). Molecular variation within and between ten populations of *Primula farinosa* (Primulaceae) along an altitudinal gradient in the northern Alps. Basic Appl. Ecol. 6, 35–45. doi: 10.1016/j.baae.2004.09.00442574925

[B61] Reisch C. Rosbakh S. (2021). Patterns of genetic variation in European plant species depend on altitude. Divers. Distrib. 27, 157–163. doi: 10.1111/ddi.1317740046247

[B62] Richards A. J. (2003). Primula. Revised Edn (Portland, OR: Timber Press).

[B500] Ronikier M. Costa A. Fuertes Aguilar J. Nieto Feliner G. Küpfer P. Mirek Z. (2008). Phylogeography of *Pulsatilla vernalis* (L.) Mill. (Ranunculaceae): chloroplast DNA reveals two evolutionary lineages across central Europe and Scandinavia. J. Biogeogr. 35, 1650–1664. doi: 10.1111/j.1365-2699.2008.01907.x

[B63] Salamon-Albert É. Morschhauser T. (2003). Habitat preferences of a unique specialist plant species (Primula farinosa subsp. alpigena) in Hungary. Acta Bot. Hung 45, 193–215. doi: 10.1556/ABot.45.2003.1-2.17

[B64] Schmitt T. (2007). Molecular biogeography of Europe: Pleistocene cycles and postglacial trends. Front. Zool. 4, 11. doi: 10.1186/1742-9994-4-11 17439649 PMC1868914

[B65] Schönswetter P. Stehlik I. Holderegger R. Tribsch A. (2005). Molecular evidence for glacial refugia of mountain plants in the European Alps. Mol. Ecol. 14, 3547–3555. doi: 10.1111/j.1365-294X.2005.02683.x 16156822

[B66] Šmídová A. Münzbergová Z. Plačková I. (2011). Genetic diversity of a relict plant species, *Ligularia sibirica* (L.) Cass. (Asteraceae). Flora 206, 151–157. doi: 10.1016/j.flora.2010.03.00342574925

[B67] Sramkó G. Molnár V. A. Hawkins J. A. Bateman R. M. (2014). Molecular phylogeny and evolutionary history of the Eurasiatic orchid genus *Himantoglossum* s.l.(Orchidaceae). Ann. Bot. 114, 1609–1626. doi: 10.1093/aob/mcu179 25294871 PMC4649687

[B68] Stewart J. R. Lister A. M. Barnes I. Dalén L. (2010). Refugia revisited: individualistic responses of species in space and time. Proc. R. Soc B. 277, 661–672. doi: 10.1098/rspb.2009.1272 19864280 PMC2842738

[B69] Theodoridis S. Randin C. F. Broennimann O. Patsiou T. S. Conti E. (2013). Divergent and narrower climatic niches characterize polyploid species of European primroses in *Primula* sect. *Aleuritia*. J. Biogeogr. 40, 1278–1289. doi: 10.1111/jbi.1208540046247

[B70] Theodoridis S. Randin C. F. Szövényi P. Boucher F. C. Patsiou T. S. Conti E. (2017). How do cold-adapted plants respond to climatic cycles? Interglacial expansion explains current distribution and genomic diversity in *Primula farinosa* L. Syst. Biol. 66, 715–736. doi: 10.1093/sysbio/syw114 28334079

[B71] Tzedakis P. C. Emerson B. C. Hewitt G. M. (2013). Cryptic or mystic? Glacial tree refugia in northern Europe. Trends Ecol. Evol. 28, 696–704. doi: 10.1016/j.tree.2013.09.001 24091207

[B72] Urbaniak J. Kwiatkowski P. (2023). The molecular population structure of *Swertia perennis* (Gentianaceae) in Central Europe. Sci. Rep. 13, 17059. doi: 10.1038/s41598-023-43731-5 37816773 PMC10564900

[B73] Weir B. S. Cockerham C. C. (1984). Estimating F-statistics for the analysis of population structure. Evolution 38, 1358–1371. doi: 10.2307/2408641 28563791

[B74] Westergaard K. B. Alsos I. G. Popp M. Engelskjøn T. Flatberg K. I. Brochmann C. (2011). Glacial survival may matter after all: nunatak signatures in the rare European populations of two west-arctic species. Mol. Ecol. 20, 376–393. doi: 10.1111/j.1365-294X.2010.04928.x 21156004

[B75] Wickham H. (2016). Ggplot2: Elegant Graphics for Data Analysis (Cham: Springer). doi: 10.1007/978-3-319-24277-4

